# Mitochondrial HMGCS1 mediates cisplatin resistance in cervical cancer through regulation of mitochondrial transcription

**DOI:** 10.1186/s12860-026-00566-y

**Published:** 2026-01-16

**Authors:** Wenxuan Yang, Shumin Liu, Dandan Shang, Ping Liu, Hongtang Shi, Hongtao Zhang, Chao Zhou

**Affiliations:** 1 Department of Obstetrics and Gynecology, Binzhou Medical University Hospital, Binzhou, Shandong 256600 P. R. China; 2 Department of Obstetrics and Gynecology, Weifang People’s Hospital, Weifang, Shandong 261000 P. R. China; 3 Department of Reproductive Medicine, Hainan Western Center Hospital, Danzhou, Hainan, 571799 P. R. China

**Keywords:** HMGCS1, Mitochondrial transcription, Cisplatin resistance, Cervical cancer, Oxidative phosphorylation

## Abstract

Cisplatin resistance remains a major challenge in the treatment of cervical cancer. Here, we identify 3-hydroxy-3-methylglutaryl-CoA synthase 1 (HMGCS1), a rate-limiting enzyme in the mevalonate pathway, as a critical regulator of cisplatin resistance through its localization to mitochondria. HMGCS1 preferentially accumulates in the mitochondria of cisplatin-resistant cervical cancer cells, and targeted expression of HMGCS1 to mitochondria, but not to the nucleus or cytosol, is sufficient to confer cisplatin resistance. Mechanistically, mitochondrial HMGCS1 associates with the D-loop region of mitochondrial DNA (mtDNA) and is required for the stable binding of the core transcription machinery components POLRMT, TFAM, and TFB2M. This regulatory function is important for mitochondrial respiratory capacity through its critical role in the transcription of mtDNA-encoded respiratory complex subunits. Genetic depletion or pharmacological inhibition of HMGCS1 disrupts mitochondrial transcription, impairs respiratory function, and re-sensitizes resistant cells to cisplatin. Moreover, combined treatment with cisplatin and inhibitors targeting either HMGCS1 or mitochondrial transcription exhibits synergistic effects against cisplatin-resistant cervical cancer cells. Our findings reveal an unexpected role for HMGCS1 as a non-canonical regulator of mitochondrial transcription and establish the HMGCS1-mitochondrial transcription axis as a promising therapeutic target to overcome cisplatin resistance in cervical cancer.

**Supplementary Information:**

The online version contains supplementary material available at 10.1186/s12860-026-00566-y.

## Introduction

Cervical cancer remains one of the most prevalent gynecological malignancies worldwide, with significant morbidity and mortality rates despite advances in prevention and treatment strategies [1, 2]. While cisplatin-based chemotherapy represents a cornerstone of treatment for advanced cervical cancer, the development of chemoresistance severely limits therapeutic efficacy and contributes to poor clinical outcomes [3]. Understanding the molecular mechanisms underlying cisplatin resistance is therefore crucial for developing more effective treatment strategies.

Mitochondria have emerged as critical organelles in cancer biology, regulating not only cellular energy production but also apoptosis, redox homeostasis, and biosynthetic processes [4–8]. Recent evidence suggests that alterations in mitochondrial function contribute significantly to chemotherapy resistance across multiple cancer types [9, 10]. Enhanced mitochondrial respiratory capacity has been associated with increased drug efflux, reduced apoptotic potential, and improved cell survival under chemotherapeutic stress [11–13]. However, the precise molecular mechanisms connecting mitochondrial metabolism to cisplatin resistance in cervical cancer remain incompletely understood.

3-Hydroxy-3-methylglutaryl-CoA synthase 1 (HMGCS1) is a rate-limiting enzyme in the mevalonate pathway, catalyzing the condensation of acetyl-CoA with acetoacetyl-CoA to form HMG-CoA [14, 15]. Traditionally, HMGCS1 has been considered a cytosolic enzyme with well-characterized metabolic functions. However, our previous studies have revealed unexpected subcellular localizations and novel functions of HMGCS1 in cervical cancer, including its presence in both nuclear and mitochondrial compartments [16]. These findings suggest that HMGCS1 may have non-canonical roles beyond its classical metabolic function. While recent studies have implicated HMGCS1 in the progression of various cancers, including colorectal, prostate, and lung cancer, the specific contributions of its compartmentalized functions to chemotherapy resistance remain poorly understood [17–20]. Particularly, the mechanistic connection between mitochondrial HMGCS1 and cisplatin resistance in cervical cancer represents a significant knowledge gap that requires investigation.

Mitochondrial DNA (mtDNA) encodes essential components of the oxidative phosphorylation system, including subunits of respiratory complexes I, III, IV, and ATP synthase [21, 22]. Transcription of mtDNA is regulated by a dedicated machinery comprising mitochondrial RNA polymerase (POLRMT), mitochondrial transcription factor A (TFAM), and mitochondrial transcription factor B2 (TFB2M), which assemble at the regulatory D-loop region [23, 24]. Alterations in mitochondrial gene expression have been linked to metabolic reprogramming in cancer cells and may contribute to therapy resistance [25, 26]. While canonical regulators of mitochondrial transcription are well-characterized, the existence of non-canonical regulators, particularly metabolic enzymes that might moonlight in this process, has been largely overlooked. Whether HMGCS1, given its unexpected mitochondrial localization, could potentially influence mitochondrial transcription and thereby modulate chemoresistance represents an entirely unexplored possibility that warrants investigation.

In this study, we investigate the mechanistic link between HMGCS1, mitochondrial metabolism, and cisplatin resistance in cervical cancer. We demonstrate that mitochondrial localization of HMGCS1 specifically drives cisplatin resistance by enhancing mitochondrial transcription and respiratory capacity. Furthermore, we identify a novel function of HMGCS1 in facilitating the assembly of the mitochondrial transcription machinery at the D-loop region, thereby promoting the expression of mtDNA-encoded respiratory complex components. Finally, we show that pharmacological inhibition of either HMGCS1 or mitochondrial transcription synergizes with cisplatin to overcome drug resistance in cervical cancer cells. These findings establish the HMGCS1-mitochondrial transcription axis as a promising therapeutic target for overcoming cisplatin resistance in cervical cancer.

## Materials and methods

### Cell culture

Cervical cancer cell lines HeLa and C33A were obtained from American Type Culture Collection (ATCC) and maintained in Dulbecco’s Modified Eagle Medium (DMEM) supplemented with 10% fetal bovine serum (FBS) and 1% penicillin/streptomycin at 37 °C with 5% CO2.

### CRISPR/Cas9-mediated HMGCS1 knockout

HMGCS1 knockout cell lines were generated using CRISPR/Cas9 technology. Two guide RNAs (gRNAs) targeting HMGCS1 (5’-ATATGTTGATCAAGCAGAGT-3’ and 5’-GCCCAATGCAATCATAGGAA-3’) were cloned into the pSpCas9(BB)-2 A-Puro (PX459) vector. Cells were transfected with the CRISPR/Cas9 constructs using Lipofectamine 3000 (Invitrogen) according to the manufacturer’s protocol. After 48 h, transfected cells were selected with 2 µg/ml puromycin for 72 h. Single cell clones were isolated by limiting dilution and validated for HMGCS1 knockout by Western blotting.

### Generation of cisplatin-resistant cell lines

Cisplatin-resistant (CisR) cell lines were established by exposing parental cells to gradually increasing concentrations of cisplatin over a period of 6 months, starting with 0.5 µM and increasing to 5 µM for HeLa and 2 µM for C33A cells. Resistant cells were maintained in medium containing 2 µM cisplatin, which was removed 72 h prior to experiments.

### Construction and expression of HMGCS1 subcellular localization vectors

Nuclear-localized HMGCS1 (NLS-HMGCS1) was generated by fusing three tandem SV40 nuclear localization signals (PKKKRKV) to the N-terminus of HMGCS1 coding sequence. Mitochondrial-targeted HMGCS1 (MTS-HMGCS1) was created by fusing the mitochondrial targeting sequence from cytochrome c oxidase subunit 8 A (COX8A, first 29 amino acids: MSVLTPLLLRGLTGSARRLPVPRAKIHSL) to the N-terminus of HMGCS1. Both constructs included a C-terminal 6×His tag for detection. The sequences were cloned into the pLVX-IRES-Puro lentiviral vector (Takara Bio). Lentiviral particles were produced in HEK293T cells using the psPAX2 and pMD2.G packaging plasmids. Target cells were transduced with lentiviral supernatants containing 8 µg/ml polybrene and selected with 2 µg/ml puromycin for 7 days.

### Cell viability and drug sensitivity assays

Cell viability was assessed using the sulforhodamine B (SRB) assay. Briefly, cells were seeded in 96-well plates (3,000 cells/well) and treated with varying concentrations of cisplatin for 72 h. After treatment, cells were fixed with 10% trichloroacetic acid for 1 h at 4 °C, washed with water, and stained with 0.057% SRB solution for 30 min. After washing with 1% acetic acid, protein-bound dye was dissolved in 10 mM Tris base, and absorbance was measured at 495 nm using a microplate reader. IC50 values were calculated using GraphPad Prism software.

### Drug synergy analysis

Drug synergy between cisplatin and hymeglusin or IMT1B was assessed using the Bliss independence model. HeLa_CisR cells were treated with various concentration combinations of cisplatin (0–60 µM) and hymeglusin (0–27 µM), while C33A_CisR cells were treated with cisplatin (0–25 µM) and hymeglusin (0–18 µM). For IMT1B combinations, HeLa_CisR cells were treated with cisplatin (0–60 µM) and IMT1B (0–10 µM), while C33A_CisR cells were treated with cisplatin (0–25 µM) and IMT1B (0–21 µM). All treatments were conducted for 72 h. Cell viability was measured using the SRB assay as described above. Drug synergy was quantified using the Bliss independence model. Dose-response matrix data were analyzed using the SynergyFinder web application (https://synergyfinder.org/). The Bliss synergy score is calculated based on the difference between the observed drug combination response (% inhibition) and the expected response calculated from the Bliss independence model, where the expected combination effect (Ebliss) is defined as Ebliss = Fa + Fb - (Fa × Fb). The final synergy score reported by the software represents the average excess response due to synergy. Positive scores indicate synergy (the observed effect is greater than the expected effect), scores near zero indicate an additive effect, and negative scores indicate antagonism.

### Subcellular fractionation and western blotting

Nuclear and mitochondrial fractions were isolated using a differential centrifugation method. Briefly, cells were harvested, washed with PBS, and resuspended in isolation buffer (225 mM mannitol, 75 mM sucrose, 0.1 mM EGTA, 30 mM Tris-HCl pH 7.4) supplemented with protease inhibitor cocktail. Cells were homogenized using a Dounce homogenizer, and the homogenate was centrifuged at 700 × g for 10 min to pellet nuclei. The supernatant was further centrifuged at 12,000 × g for 15 min to isolate mitochondria. The nuclear pellet was washed twice with isolation buffer before lysis. Mitochondrial pellets were washed twice with isolation buffer to minimize cytosolic contamination. Both nuclear and mitochondrial pellets were lysed in RIPA buffer (50 mM Tris-HCl pH 8.0, 150 mM NaCl, 1% NP-40, 0.5% sodium deoxycholate, 0.1% SDS) containing protease inhibitor cocktail. Protein concentrations were determined using the BCA Protein Assay Kit (Pierce).

For Western blotting, protein samples (20 µg) were separated by SDS-PAGE and transferred to PVDF membranes. After blocking with 5% non-fat milk, membranes were incubated with primary antibodies against HMGCS1 (1:1000, Abcam, ab194971), Lamin B1 (1:1000, Abcam, ab16048), TOMM20 (1:1000, Santa Cruz, sc-17764), or TUBA1A (1:5000, Sigma, T9026) overnight at 4 °C. After washing, membranes were incubated with HRP-conjugated secondary antibodies (1:5000) for 1 h at room temperature. Protein bands were visualized using ECL reagent (Pierce).

### Immunofluorescence microscopy

Cells grown on coverslips were fixed with 4% paraformaldehyde for 15 min, permeabilized with 0.2% Triton X-100 for 10 min, and blocked with 3% BSA for 1 h. For mitochondrial staining, cells were incubated with 200 nM MitoTracker Red CMXRos (Invitrogen) for 30 min prior to fixation. Fixed cells were incubated with anti-6×His antibody (1:200, Cell Signaling, #2365) overnight at 4 °C, followed by Alexa Fluor 488-conjugated secondary antibody (1:500, Invitrogen) for 1 h at room temperature. Nuclei were stained with DAPI. Images were acquired using a Zeiss LSM 880 confocal microscope and analyzed using ZEN software.

### Metabolic analysis using seahorse XF analyzer

Mitochondrial respiration was measured using the Seahorse XFe96 Analyzer (Agilent Technologies). Cells were seeded in XF96 cell culture microplates at 2 × 10^4^ cells/well and incubated overnight. Before the assay, culture medium was replaced with XF base medium supplemented with 1 mM pyruvate, 2 mM glutamine, and 10 mM glucose (pH 7.4). The Seahorse XF Cell Mito Stress Test was performed according to the manufacturer’s protocol, with sequential injections of oligomycin (1 µM), FCCP (1 µM), and rotenone/antimycin A (0.5 µM each). Oxygen consumption rate (OCR) was normalized to protein concentration determined by BCA Protein Assay Kit (Pierce) after the assay. Basal respiration was calculated as the difference between baseline OCR and non-mitochondrial respiration. Maximal respiration was calculated as the difference between FCCP-stimulated OCR and non-mitochondrial respiration.

### Cholesterol quantification

Intracellular cholesterol levels were measured using the Cholesterol/Cholesteryl Ester Quantitation Assay Kit (Abcam, ab65359) according to the manufacturer’s instructions. Briefly, cells (1 × 10^6^) were washed with cold PBS and lipids were extracted by resuspending cell pellets in 200 µL of chloroform: isopropanol: NP-40 (7:11:0.1) using a micro-homogenizer. The extract was centrifuged at 15,000 × g for 10 min, and the entire liquid (organic phase) was transferred to a new tube, avoiding the pellet. Samples were air-dried at 50 °C to remove chloroform, followed by vacuum drying for 30 min to remove trace organic solvent. The dried lipids were dissolved in 200 µL of Cholesterol Assay Buffer by sonication or vortexing. For the assay, 50 µL of each sample was transferred to a 96-well plate. To measure total cholesterol (free cholesterol + cholesteryl esters), 50 µL of Total Cholesterol Reaction Mix containing Assay Buffer, Cholesterol Probe, Enzyme Mix, and Cholesterol Esterase was added to wells. To measure free cholesterol only, 50 µL of Free Cholesterol Reaction Mix (without Cholesterol Esterase) was added. The plate was incubated at 37 °C for 60 min protected from light, and absorbance was measured at 570 nm.

### ROS measurement

Intracellular reactive oxygen species (ROS) levels were measured using the Cellular Reactive Oxygen Species Detection Assay Kit (Deep Red Fluorescence) (Abcam, ab186029) according to the manufacturer’s protocol. Cells were seeded in black-walled, clear-bottom 96-well plates at 2 × 10^4^ cells per well in 90 µL growth medium and incubated overnight. The next day, cells were treated with test compounds for the desired duration. ROS Deep Red working solution was prepared by adding 20 µL of 1000X ROS Deep Red stock solution to 10 mL of assay buffer. Following treatment, 100 µL of ROS Deep Red working solution was added to each well, and cells were incubated at 37 °C in a 5% CO₂ incubator for 30 min. Fluorescence intensity was measured using a microplate reader at Ex/Em = 650/675 nm with bottom read mode.

### RNA extraction and quantitative real-time PCR

Total RNA was isolated using the RNeasy Mini Kit (Qiagen) according to the manufacturer’s instructions. RNA concentration and purity were determined using a NanoDrop spectrophotometer. For cDNA synthesis, 1 µg of total RNA was reverse transcribed using the SuperScript IV First-Strand Synthesis System (Invitrogen). Quantitative PCR was performed using the PowerUp SYBR Green Master Mix (Applied Biosystems) on a QuantStudio 6 Flex Real-Time PCR System (Applied Biosystems). Primer sequences for mitochondrial genes and reference genes are listed in Supplementary Table 1. Relative gene expression was calculated using the 2^−ΔΔCt^ method, with ACTB as the reference gene.

### Mitochondrial DNA copy number analysis

Total DNA was extracted using the DNeasy Blood & Tissue Kit (Qiagen) according to the manufacturer’s protocol. Mitochondrial DNA (mtDNA) copy number was determined by qPCR using primers for MT-ND1 (mitochondrial gene) and B2M (nuclear gene). The ratio of MT-ND1 to B2M was calculated to represent the relative mtDNA copy number. Primer sequences are listed in Supplementary Table 1.

### RNA synthesis and decay assays

For RNA synthesis assay, cells were pulse-labeled with 5-ethynyl uridine (EU) using the Click-iT RNA Alexa Fluor 488 Imaging Kit (Invitrogen) with modifications for biochemical analysis. Briefly, cells were incubated with 1 mM EU for 0 (T0) or 2 h (T1). Total RNA was isolated, and EU-labeled RNA was captured using the Click-iT Nascent RNA Capture Kit (Invitrogen). For RNA decay assay, cells were treated with 2 µg/ml actinomycin D (to inhibit nuclear transcription) and 1 µM IMT1B (to inhibit mitochondrial transcription). RNA was isolated at 0 (T0) and 24 h (T1) after inhibitor treatment. Captured RNA from synthesis assays and total RNA from decay assays were reverse transcribed and analyzed by qPCR as described above. Primer sequences for all transcripts are listed in Supplementary Table 1.

### Mitochondrial DNA immunoprecipitation (MtDIP)

MtDIP was performed using a modified chromatin immunoprecipitation protocol. Briefly, cells (5 × 10^6^) were crosslinked with 1% formaldehyde for 10 min at room temperature, followed by quenching with 125 mM glycine. Cells were lysed, and mitochondria were isolated as described above. Mitochondrial lysates were sonicated to shear mtDNA to an average size of 200–500 bp. After pre-clearing with Protein A/G agarose beads, lysates were incubated with antibodies against HMGCS1 (Abcam, ab194971), POLRMT (Abcam, ab228576), TFAM (Cell Signaling, #8076), or TFB2M (Abcam, ab228614) overnight at 4 °C. Immune complexes were captured with Protein A/G agarose beads, washed extensively, and eluted. Crosslinks were reversed by heating at 65 °C overnight. DNA was purified using the QIAquick PCR Purification Kit (Qiagen) and analyzed by qPCR using primers specific for different regions of mtDNA, including various D-loop regions and mitochondrial genes. Primer sequences are listed in Supplementary Table 1. Results were calculated as a percentage of input DNA.

### Statistical analysis

All experiments were performed at least three times independently. Data are presented as mean ± standard deviation (SD). Statistical analysis was performed using GraphPad Prism 9.0 software. Differences between two groups were analyzed using Student’s t-test. For comparisons involving more than two groups for a single dependent variable (e.g., respiration and ROS levels), One-way ANOVA followed by Dunnett’s multiple comparisons test was used. For comparisons involving two independent variables (e.g., gene expression across different cell groups), Two-way ANOVA followed by Dunnett’s multiple comparisons test was performed to compare column means within each row. For dose-response curves, IC50 values were determined by nonlinear regression (four parameters, variable slope), and the statistical significance of differences between LogIC50 values was assessed using the Extra sum-of-squares F-test, with a Bonferroni correction applied for multiple pairwise comparisons. P values < 0.05 were considered statistically significant.

## Results

### HMGCS1 mediates cisplatin resistance in cervical cancer through mitochondrial accumulation

Our previous study has established that HMGCS1 regulates mitochondrial function, and mitochondrial respiratory activity is known to influence radiotherapy outcomes [16]. We therefore investigated whether HMGCS1 similarly affects cervical cancer cell sensitivity to cisplatin chemotherapy.

However, before assessing its role in drug response, we needed to address findings from other cancer contexts where HMGCS1 depletion was shown to negatively impact cell viability [27, 28]. To rule out this potential confounding factor in our system, we first assessed the effect of HMGCS1 knockout on the basal proliferation of HeLa and C33A cells. Interestingly, we found that HMGCS1 knockout did not significantly alter the proliferation rate of either cervical cancer cell line (Supplementary Fig. S1A-B). This key finding suggests the role of HMGCS1 in proliferation is context-dependent and, crucially, allows us to interpret any subsequent changes in drug sensitivity as a specific effect, rather than a consequence of generally compromised cell fitness.

**Fig. 1:**
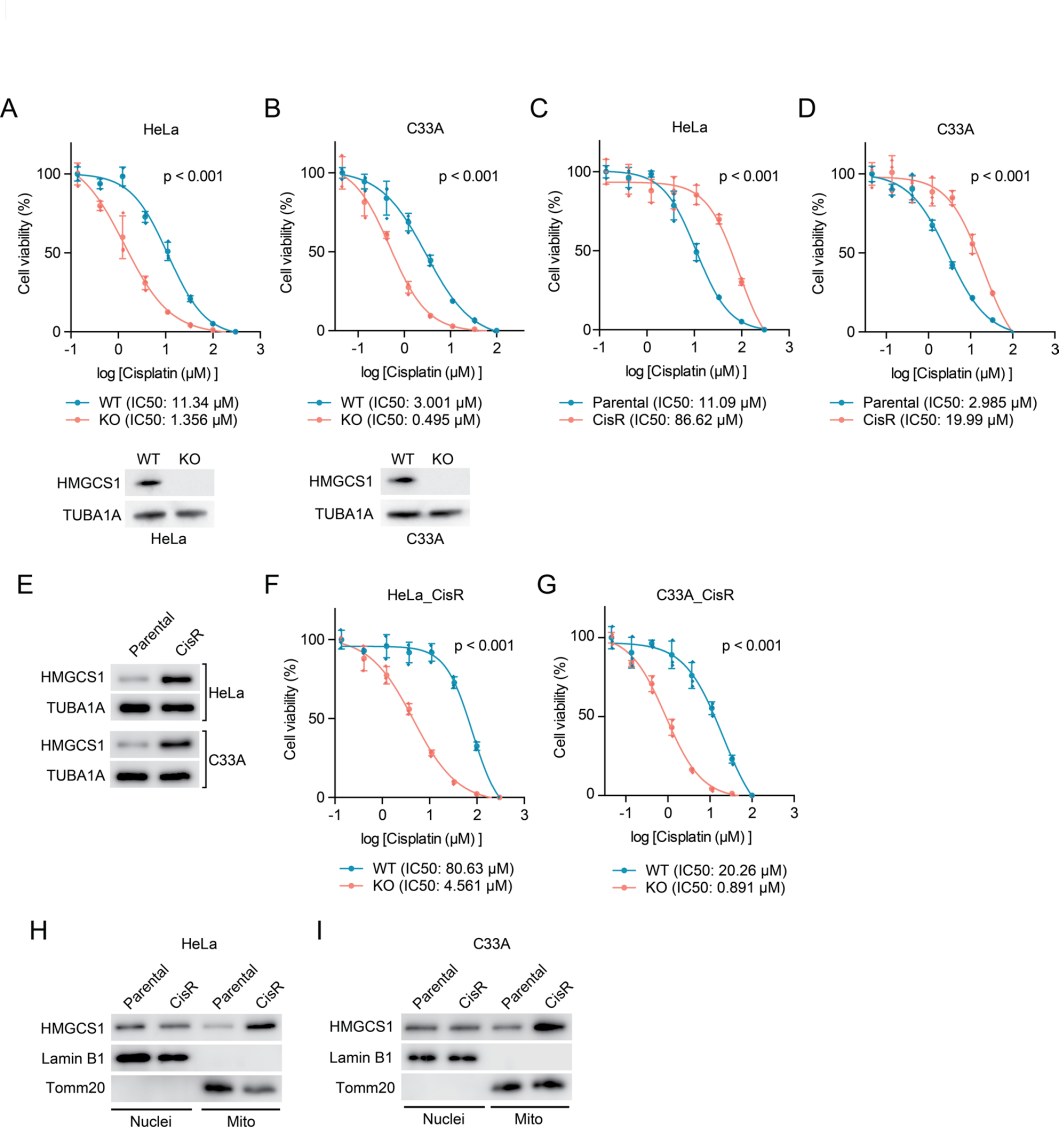
HMGCS1 regulates cisplatin sensitivity and resistance in cervical cancer cells through mitochondrial localization. (**A**-**B**) Dose-response curves showing cell viability of wild-type (WT) and HMGCS1 knockout (KO) HeLa (**A**) and C33A (**B**) cells treated with increasing concentrations of cisplatin for 72 h. IC50 values are indicated. Cell viability was determined using SRB assay. The corresponding Western blots below validate HMGCS1 protein knockout, using TUBA1A as a loading control. (**C**-**D**) Dose-response curves showing cell viability of parental and cisplatin-resistant (CisR) HeLa (**C**) and C33A (**D**) cells treated with increasing concentrations of cisplatin for 72 h. IC50 values are indicated. (**E**) Western blot analysis of HMGCS1 protein expression in parental and cisplatin-resistant HeLa and C33A cells. TUBA1A was used as a loading control. (**F**-**G**) Dose-response curves showing cell viability of wild-type (WT) and HMGCS1 knockout (KO) cisplatin-resistant HeLa_CisR (**F**) and C33A_CisR (**G**) cells treated with increasing concentrations of cisplatin for 72 h. IC50 values are indicated. (**H**-**I**) Western blot analysis of HMGCS1 protein expression in nuclear and mitochondrial fractions of parental and cisplatin-resistant HeLa (**H**) and C33A (**I**) cells. Lamin B1 and Tomm20 were used as nuclear and mitochondrial markers, respectively. (**A**-**D**, **F**-**G**) Data are presented as mean ± SD. Statistical significance of the difference between IC50 values was determined by the Extra Sum-of-Squares F-test

With this established, we next examined the effect of HMGCS1 depletion on cisplatin response. Knockout of HMGCS1 in both HeLa and C33A cervical cancer cell lines significantly enhanced cisplatin sensitivity, as demonstrated by dose-response curves (Fig. 1A-B). IC50 values decreased by more than 6-fold in HMGCS1-KO cells compared to wild-type controls in both cell lines. To further explore this relationship, we established cisplatin-resistant (CisR) HeLa and C33A cell lines through long-term drug exposure. SRB assays confirmed the resistant phenotype, with significantly higher IC50 values in resistant cells compared to their parental counterparts (Fig. 1C-D). Notably, Western blot analysis revealed markedly elevated HMGCS1 protein levels in cisplatin-resistant cells relative to parental cells (Fig. 1E), suggesting a potential correlation between HMGCS1 upregulation and acquired resistance to cisplatin in cervical cancer cells.

To determine whether HMGCS1 plays a causal role in cisplatin resistance, we knocked out HMGCS1 in the established cisplatin-resistant cell lines. HMGCS1 knockout significantly re-sensitized resistant cells to cisplatin treatment (Fig. 1F-G), with IC50 values decreasing by more than 17-fold in both resistant cell lines. Given this critical role of HMGCS1 in cisplatin resistance, we next investigated the mechanism behind its function. Since our previous research had demonstrated that HMGCS1 is localized in both the nucleus and mitochondria of cervical cancer cells [16], we sought to determine whether the subcellular distribution of HMGCS1 might be altered in resistant cells. Cell fractionation followed by Western blot analysis revealed a striking pattern: while total HMGCS1 levels were elevated in resistant cells, this increase was specifically attributable to mitochondrial HMGCS1 accumulation (Fig. 1H-I). No significant differences in nuclear HMGCS1 levels were observed between resistant and parental cell lines (Fig. 1H-I). This compartment-specific accumulation suggests that mitochondrial, rather than nuclear, HMGCS1 may be the primary mediator of cisplatin resistance in cervical cancer cells.

### Mitochondrial HMGCS1 is essential for cisplatin resistance in cervical cancer cells

**Fig. 2:**
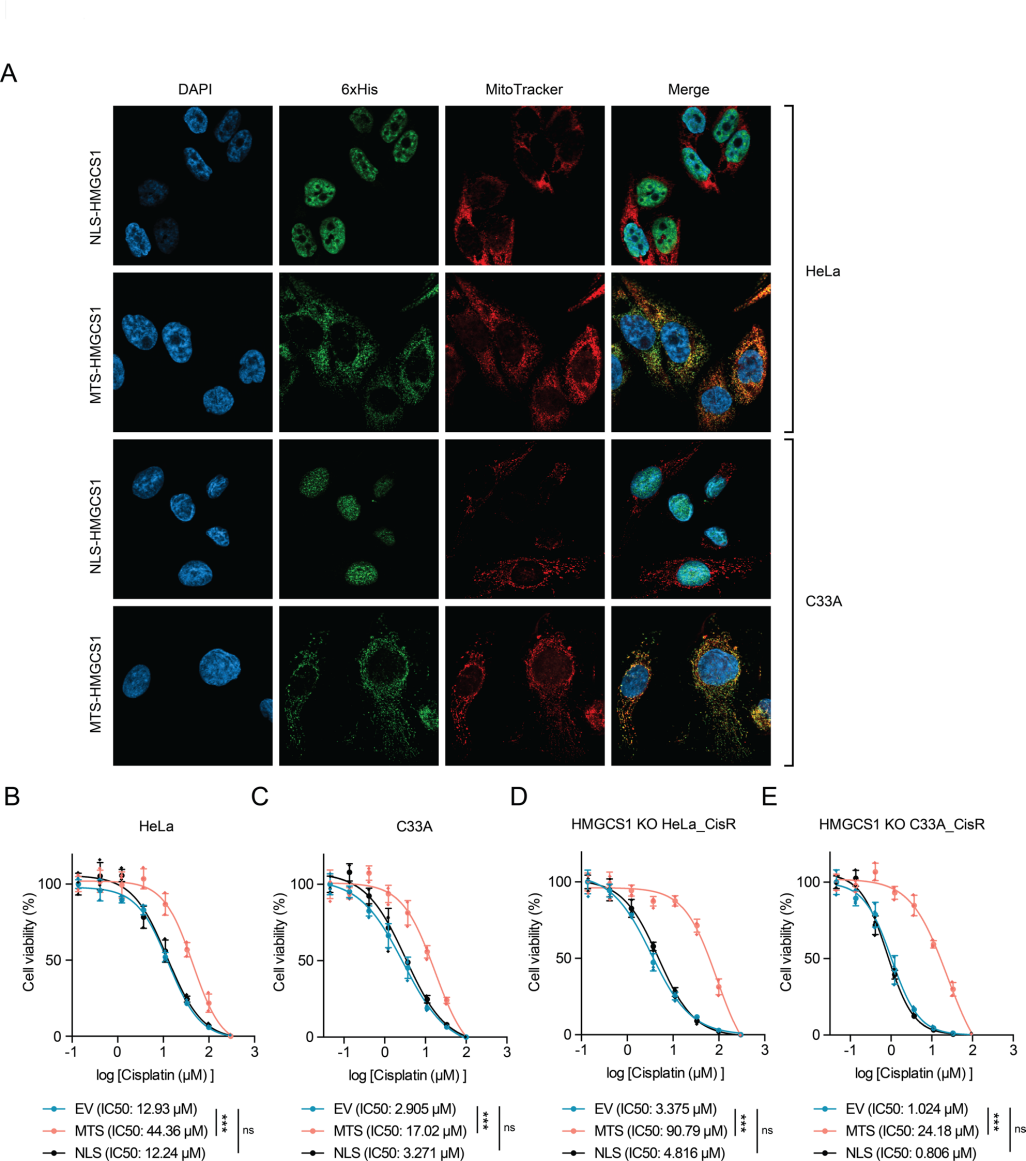
Subcellular localization of HMGCS1 determines its role in cisplatin resistance. (**A**) Immunofluorescence microscopy of HeLa and C33A cells expressing 6xHis-tagged NLS-HMGCS1 or MTS-HMGCS1. Cells were stained with DAPI (blue) for nuclei, anti-6xHis antibody (green) for tagged HMGCS1, and MitoTracker (red) for mitochondria. Merged images show the distinct subcellular localization of each construct. (**B-E**) Dose-response curves showing cell viability after cisplatin treatment (72 h) in: parental HeLa (**B**) and C33A (**C**) cells; and HMGCS1-knockout cisplatin-resistant HeLa_CisR (**D**) and C33A_CisR (**E**) cells expressing empty vector (EV), MTS-HMGCS1, or NLS-HMGCS1. Cell viability was determined using SRB assay. Data are presented as mean ± SD. IC50 values are indicated. Statistical significance was determined by pairwise comparison of the LogIC50 value for each construct against the EV control using the Extra sum-of-squares F-test, with a Bonferroni correction for multiple comparisons (***p < 0.001, ns: not significant)

To further validate whether mitochondrial HMGCS1 specifically mediates cisplatin resistance, we designed constructs to selectively increase HMGCS1 levels in distinct subcellular compartments. We generated 6xHis-tagged HMGCS1 with either a nuclear localization signal (NLS-HMGCS1) or a mitochondrial targeting sequence (MTS-HMGCS1), and introduced these constructs into parental HeLa and C33A cells. To validate these expression constructs, we first confirmed by Western blotting that the MTS-HMGCS1 and NLS-HMGCS1 proteins were expressed at comparable levels in total cell lysates (Supplementary Fig. S1C-D). Next, we confirmed their correct subcellular localization. As shown by immunofluorescence microscopy, NLS-HMGCS1 colocalized with DAPI-stained nuclei, while MTS-HMGCS1 colocalized with MitoTracker-labeled mitochondria (Fig. 2A). This specific targeting was further corroborated by subcellular fractionation and Western blot analysis, which verified the enrichment of NLS-HMGCS1 in the nucleus and MTS-HMGCS1 in the mitochondria (Supplementary Fig. S1E).

Before assessing their impact on drug sensitivity, we verified that expression of these constructs did not alter basal cell proliferation. Neither MTS-HMGCS1 nor NLS-HMGCS1 expression affected the proliferation rate in WT or HMGCS1-KO cells compared to empty vector controls (Supplementary Fig. S1F-I). This comprehensive validation ensures that any observed effects on cisplatin sensitivity are due to the specific functions of compartmentalized HMGCS1, rather than baseline changes in cell growth or differences in protein expression.

We next examined the functional impact of compartment-specific HMGCS1 overexpression on cisplatin sensitivity. Remarkably, cells expressing MTS-HMGCS1 exhibited significantly increased IC50 values for cisplatin compared to empty vector controls in both HeLa and C33A cells (Fig. 2B-C). In contrast, cells expressing NLS-HMGCS1 showed no significant change in cisplatin sensitivity, with IC50 values comparable to those of control cells (Fig. 2B-C). These findings indicate that only mitochondrial, not nuclear, accumulation of HMGCS1 contributes to cisplatin resistance in cervical cancer cells.

To further substantiate this conclusion, we performed rescue experiments using the HMGCS1-knockout cisplatin-resistant cell lines we had established. We introduced either MTS-HMGCS1 or NLS-HMGCS1 into these cells and assessed their cisplatin sensitivity. Consistent with our hypothesis, expression of MTS-HMGCS1 significantly restored cisplatin resistance in both HeLa_CisR and C33A_CisR HMGCS1-KO cells, increasing IC50 values in both HeLa_CisR and C33A_CisR cells (Fig. 2D-E). In contrast, expression of NLS-HMGCS1 failed to rescue cisplatin resistance in either cell line, with IC50 values remaining similar to those of empty vector controls.

Collectively, these results provide strong evidence that mitochondrial, rather than nuclear, HMGCS1 is the critical mediator of cisplatin resistance in cervical cancer cells.

### HMGCS1-mediated enhancement of mitochondrial respiration is associated with cisplatin resistance in cervical cancer

**Fig. 3:**
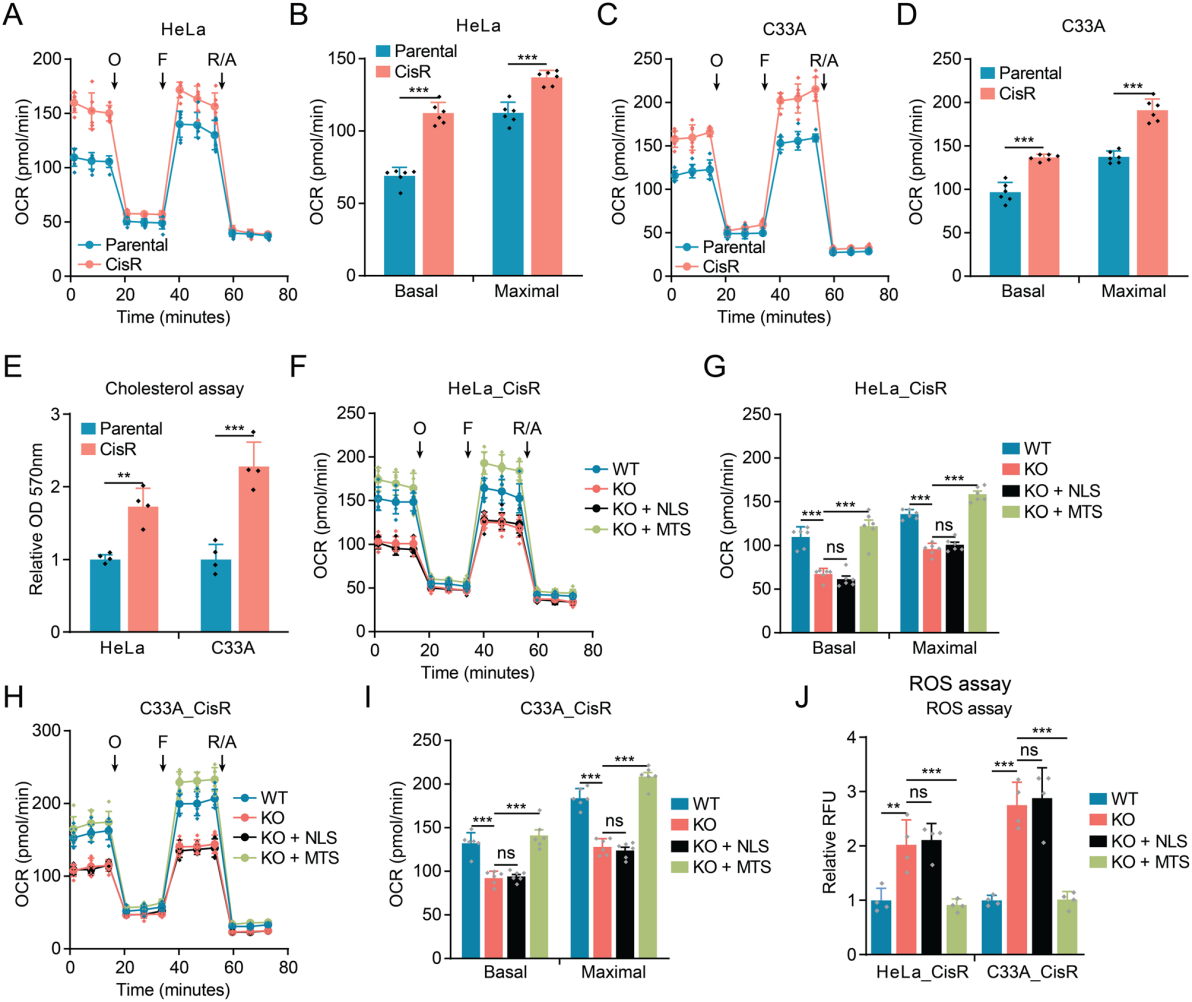
Mitochondrial HMGCS1 enhances oxidative phosphorylation and regulates redox homeostasis in cisplatin-resistant cervical cancer cells. (**A**, **C**) Mitochondrial respiration profiles showing oxygen consumption rate (OCR) in parental and cisplatin-resistant (CisR) HeLa (**A**) and C33A (**C**) cells. Sequential addition of oligomycin (O), FCCP (F), and rotenone/antimycin A (R/A) is indicated by arrows. (**B**, **D**) Quantification of basal and maximal respiratory capacity in parental and cisplatin-resistant HeLa (**B**) and C33A (**D**) cells. (**E**) Relative intracellular cholesterol levels in parental and cisplatin-resistant HeLa and C33A cells as measured by a commercial cholesterol assay kit. (**F**, **H**) Mitochondrial respiration profiles of wild-type (WT), HMGCS1-knockout (KO), and KO cells expressing NLS-HMGCS1 (KO + NLS) or MTS-HMGCS1 (KO + MTS) in HeLa_CisR (**F**) and C33A_CisR (**H**) cells. (**G**, **I**) Quantification of basal and maximal respiratory capacity in WT, KO, and rescued HeLa_CisR (**G**) and C33A_CisR (**I**) cells. (**J)** Relative ROS levels in wild-type, HMGCS1-knockout, and rescued HeLa_CisR and C33A_CisR cells measured by measured by ROS Deep Red fluorescence. Data are presented as mean ± SD. Statistical significance for panels **G**, **I**, and **J** was determined by One-way ANOVA with Dunnett’s multiple comparisons test. Comparisons in panels **B**, **D**, and **E** were made using Student’s t-test: **p < 0.01, ***p < 0.001, ns: not significant, by Student’s t-test

Given our previous findings that mitochondrial HMGCS1 modulates mitochondrial respiratory function in cervical cancer [16] and the established role of mitochondrial metabolism in chemotherapy resistance [10, 29], we hypothesized that HMGCS1 might confer cisplatin resistance by enhancing mitochondrial respiration. To test this hypothesis, we first compared mitochondrial respiratory capacity between cisplatin-resistant and parental cervical cancer cells using Seahorse XF analyzer. The oxygen consumption rate (OCR) measurements revealed significantly elevated basal and maximal respiration in cisplatin-resistant cells compared to their parental counterparts in both HeLa and C33A cell lines (Fig. 3A-D). These results indicate that enhanced mitochondrial respiratory capacity is associated with cisplatin resistance in cervical cancer cells.

Since HMGCS1 is a key enzyme in the mevalonate pathway, which produces cholesterol and other isoprenoids [30, 31], we measured intracellular cholesterol levels in resistant and parental cells. ELISA analysis revealed significantly elevated cholesterol content in cisplatin-resistant cells compared to parental cells in both HeLa and C33A lines (Fig. 3E), suggesting a potential link between cholesterol metabolism and cisplatin resistance.

To establish a causal relationship between mitochondrial HMGCS1, respiratory function, and cisplatin resistance, we examined how HMGCS1 knockout affected mitochondrial respiration in resistant cells. HMGCS1 depletion in cisplatin-resistant cells significantly decreased both basal and maximal respiration in HeLa_CisR and C33A_CisR cells (Fig. 3F-I). Importantly, expression of MTS-HMGCS1, but not NLS-HMGCS1, restored mitochondrial respiratory capacity in HMGCS1-knockout resistant cells to levels comparable to those in wild-type resistant cells (Fig. 3F-I). These findings demonstrate that mitochondrial HMGCS1 is necessary and sufficient for the enhanced mitochondrial respiration observed in cisplatin-resistant cells.

Mitochondrial dysfunction is often associated with increased reactive oxygen species (ROS) production, which can sensitize cancer cells to chemotherapy [32, 33]. We therefore measured ROS levels in our cell lines and found that HMGCS1 knockout significantly increased ROS production in both HeLa_CisR and C33A_CisR cells (Fig. 3J). Consistent with our previous observations, introduction of MTS-HMGCS1, but not NLS-HMGCS1, restored ROS levels in HMGCS1-knockout resistant cells (Fig. 3J). NLS-HMGCS1 expression had no effect on ROS levels, further supporting the specific role of mitochondrial HMGCS1 in maintaining redox homeostasis (Fig. 3J).

Collectively, these results support a mechanistic link between mitochondrial HMGCS1, enhanced mitochondrial respiration, cholesterol metabolism, and cisplatin resistance in cervical cancer cells. These findings suggest that mitochondrial HMGCS1 contributes to cisplatin resistance, a phenotype that is associated with enhanced mitochondrial respiratory capacity and maintained redox homeostasis.

### Mitochondrial HMGCS1 regulates MtDNA expression without affecting MtDNA copy number in cisplatin-resistant cervical cancer cells

**Fig. 4:**
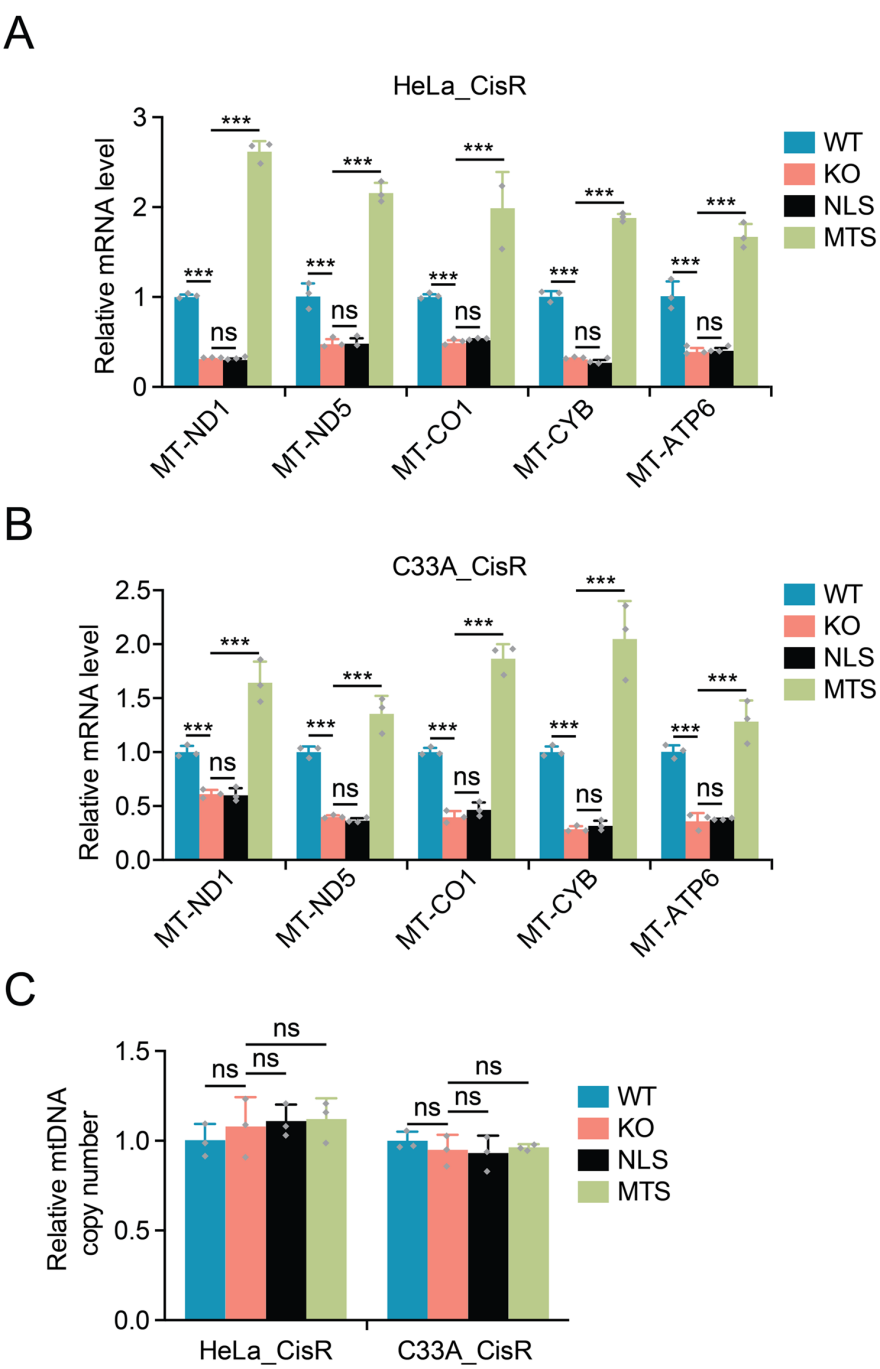
Mitochondrial HMGCS1 enhances mtDNA-encoded gene expression without affecting mtDNA copy number in cisplatin-resistant cells. (**A**-**B**) Relative mRNA expression levels of mtDNA-encoded respiratory complex genes (MT-ND1, MT-ND5, MT-CO1, MT-CYB, and MT-ATP6) in HeLa_CisR (**A**) and C33A_CisR (**B**) cells with indicated HMGCS1 status: wild-type (WT), knockout (KO), nuclear-localized HMGCS1 (NLS), or mitochondria-targeted HMGCS1 (MTS). (**C**) Relative mtDNA copy number quantification in HeLa_CisR and C33A_CisR cells with different HMGCS1 status. Data are presented as mean ± SD. Statistical significance was determined using Two-way ANOVA (**A**, **B**) or One-way ANOVA (**C**), followed by Dunnett’s multiple comparisons test: ***p < 0.001, ns: not significant

Our previous studies had established that HMGCS1 regulates mtDNA expression levels in cervical cancer cells [16], prompting us to examine whether a similar regulatory mechanism operates in cisplatin-resistant cells. We first analyzed the expression of key mitochondrial genes encoded by mtDNA in wild-type, HMGCS1-knockout, and rescue cells. HMGCS1 knockout significantly reduced the mRNA expression of mtDNA-encoded respiratory genes in both HeLa_CisR and C33A_CisR cells (Fig. 4A and B). The affected genes included MT-ND1 and MT-ND5 (Complex I components), MT-CO1 (Complex IV component), MT-CYB (Complex III component), and MT-ATP6 (ATP synthase component).

Re-expression of mitochondria-targeted HMGCS1 (MTS-HMGCS1) not only restored but significantly enhanced the expression of these mtDNA-encoded genes in both cell lines (Fig. 4A and B). In contrast, nuclear-targeted HMGCS1 (NLS-HMGCS1) failed to rescue mtDNA gene expression in HMGCS1-knockout cells, with expression levels remaining comparable to those in knockout cells (Fig. 4A and B). These results confirm that the mitochondrial localization of HMGCS1 is crucial for regulating mtDNA expression.

To determine whether the changes in mtDNA gene expression were due to alterations in mtDNA content, we quantified mtDNA copy number in our cell lines. Interestingly, neither HMGCS1 knockout nor the expression of MTS-HMGCS1 or NLS-HMGCS1 significantly affected mtDNA copy number in HeLa_CisR or C33A_CisR cells (Fig. 4C). This finding indicates that HMGCS1 specifically regulates mtDNA transcription or transcript stability rather than mtDNA replication or maintenance.

### HMGCS1 deficiency impairs mitochondrial RNA synthesis without affecting RNA stability

**Fig. 5:**
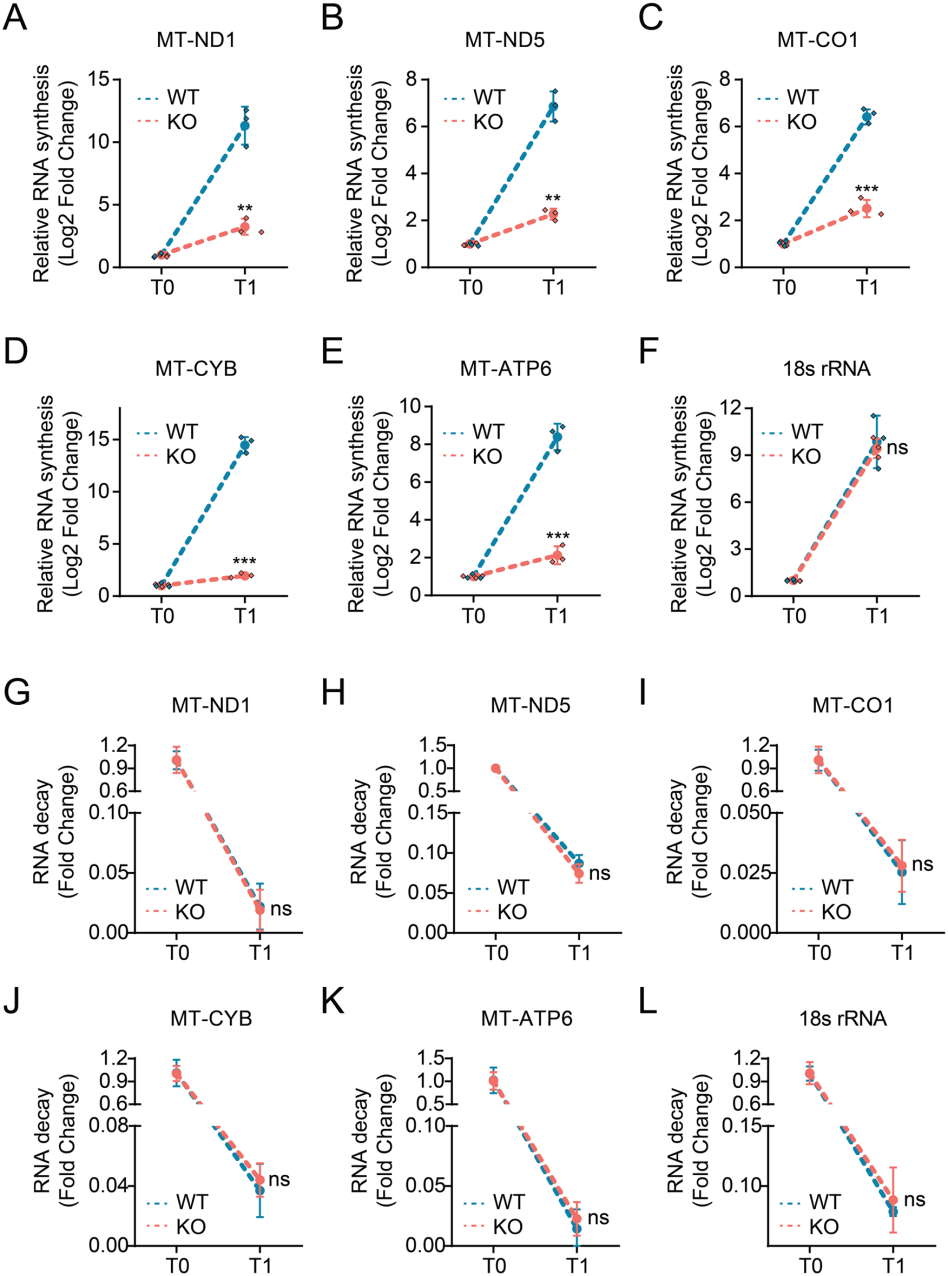
HMGCS1 regulates mitochondrial gene expression by affecting RNA synthesis rather than RNA stability. (**A**-**F**) RNA synthesis assay measuring relative accumulation of newly synthesized transcripts in wild-type (WT) and HMGCS1-knockout (KO) HeLa_CisR cells at initial (T0) and 2-hour (T1) time points for mtDNA-encoded genes (MT-ND1, MT-ND5, MT-CO1, MT-CYB, and MT-ATP6) and nuclear-encoded 18 S rRNA control. (**G**-**L**) RNA decay assay measuring the stability of the same transcripts in WT and KO HeLa_CisR cells following transcription inhibition. RNA levels were measured immediately (T0) and 24 h (T1) after adding transcription inhibitors. Data are presented as mean ± SD. Statistical significance was determined using Student’s t-test: **p < 0.01, ***p < 0.001, ns: not significant

Next, we investigated the molecular mechanism by which HMGCS1 regulates mtDNA gene expression. We hypothesized that HMGCS1 could influence mtDNA expression either by modulating mtRNA synthesis or by affecting mtRNA stability. To distinguish between these possibilities, we performed RNA synthesis and decay assays for mtDNA-encoded transcripts in HeLa_CisR cells with or without HMGCS1. For RNA synthesis measurements, we labeled newly synthesized RNA and quantified the accumulation of mtDNA-encoded transcripts over time. As shown in Fig. 5A-E, HMGCS1 knockout cells exhibited significantly reduced synthesis rates for all tested mtDNA-encoded transcripts (MT-ND1, MT-ND5, MT-CO1, MT-CYB, and MT-ATP6) compared to wild-type cells. In contrast, the synthesis of nuclear-encoded 18 S rRNA was unaffected by HMGCS1 status (Fig. 5F), confirming the specificity of HMGCS1’s effect on mitochondrial gene transcription.

To determine whether HMGCS1 also influences mtRNA stability, we measured the decay rates of mtDNA-encoded transcripts. Interestingly, RNA decay assays revealed no significant differences in the degradation rates of mtDNA-encoded transcripts between wild-type and HMGCS1 knockout cells (Fig. 5G-K). Similarly, the decay rate of nuclear-encoded 18 S rRNA was unaffected (Fig. 5L). These results indicate that HMGCS1 does not regulate mtRNA stability. Collectively, these findings demonstrate that mitochondrial HMGCS1 specifically enhances mtDNA transcription without affecting mtRNA stability.

To directly correlate this regulatory mechanism with the cisplatin-resistant phenotype, we performed the same assays comparing parental and cisplatin-resistant cells. Consistent with our findings, the cisplatin-resistant cells, which have higher levels of mitochondrial HMGCS1, displayed a significantly elevated rate of mitochondrial RNA synthesis compared to the parental cells, with no changes observed in RNA stability (Supplementary Fig. S2). This provides a direct link between the resistance phenotype and the HMGCS1-mediated enhancement of mitochondrial transcription.

### HMGCS1 facilitates mitochondrial transcription by promoting the assembly of transcription machinery at the D-loop region

**Fig. 6:**
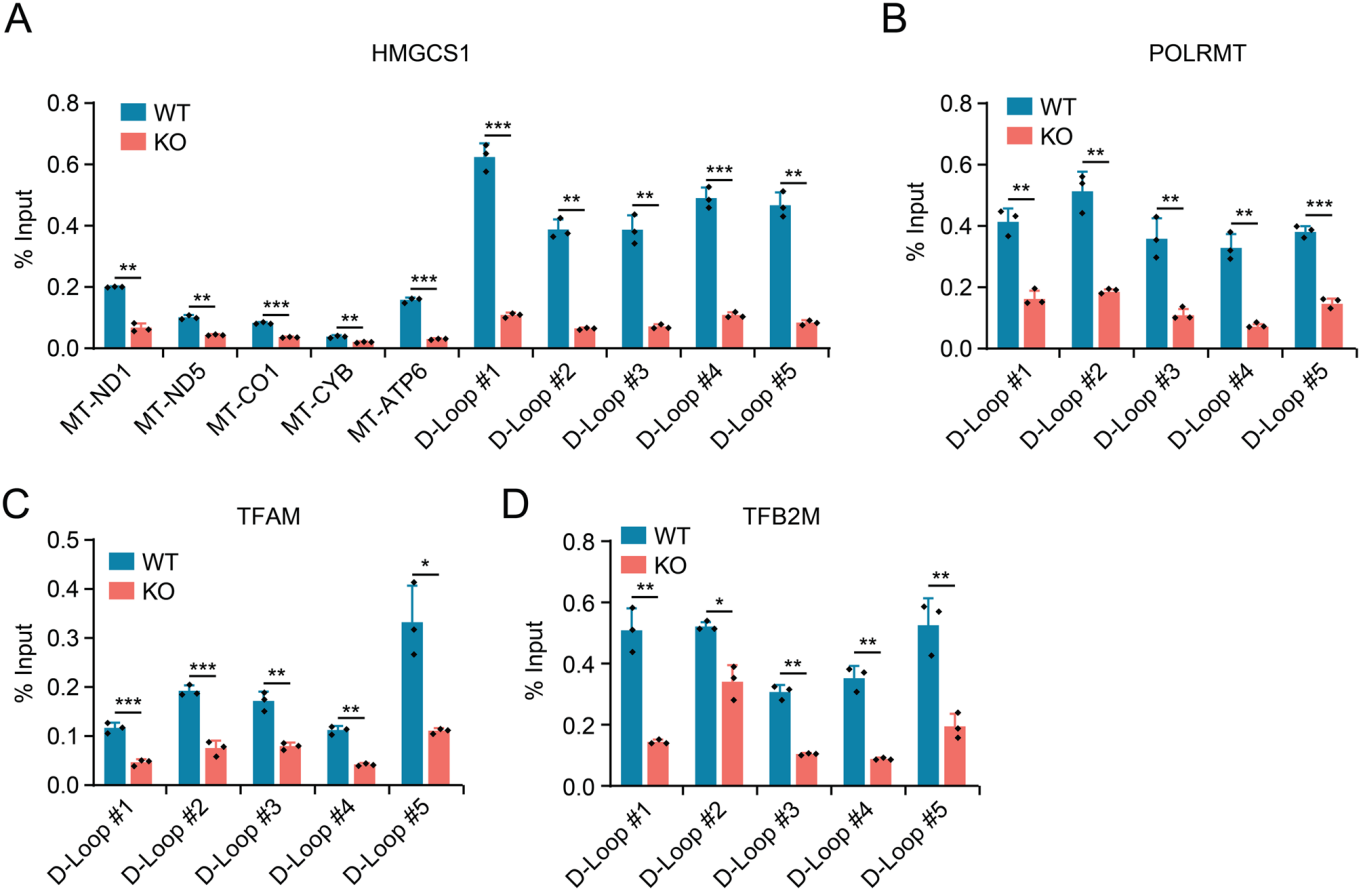
HMGCS1 binds to mtDNA D-loop region and facilitates assembly of the mitochondrial transcription complex. Mitochondrial DNA immunoprecipitation (MtDIP) analysis showing the binding of (**A**) HMGCS1, (**B**) mitochondrial RNA polymerase (POLRMT), (**C**) mitochondrial transcription factor A (TFAM), and (**D**) mitochondrial transcription factor B2 (TFB2M) to different regions of mtDNA in wild-type (WT) and HMGCS1-knockout (KO) HeLa_CisR cells. The binding enrichment is presented as percentage of input. Results for mtDNA-encoded genes (MT-ND1, MT-ND5, MT-CO1, MT-CYB, MT-ATP6) and five regions of the regulatory D-loop are shown. Data are presented as mean ± SD. Statistical significance was determined using Student’s t-test: *p < 0.05, **p < 0.01, ***p < 0.001

To further elucidate the molecular mechanism by which HMGCS1 influences mtDNA transcription, we investigated the physical interaction between HMGCS1 and mtDNA using mitochondrial DNA immunoprecipitation (MtDIP). This approach allowed us to assess the binding pattern of HMGCS1 across different regions of the mitochondrial genome. Interestingly, our MtDIP analysis revealed that HMGCS1 was highly enriched at the mitochondrial D-loop region, which contains the major regulatory elements for mtDNA transcription (Fig. 6A). HMGCS1 exhibited particularly strong binding to the D-loop regions #1–5, with significantly higher enrichment compared to coding regions (MT-ND1, MT-ND5, MT-CO1, MT-CYB, and MT-ATP6). This preferential localization suggests that HMGCS1 might participate in the regulation of mtDNA transcription initiation.

Given this observation, we hypothesized that HMGCS1 could influence the assembly or stability of the mitochondrial transcription machinery at the D-loop region. To test this possibility, we performed MtDIP experiments targeting key components of the mitochondrial transcription complex, including mitochondrial RNA polymerase (POLRMT), mitochondrial transcription factor A (TFAM), and mitochondrial transcription factor B2 (TFB2M) in control and HMGCS1-knockout cells. Strikingly, the binding of POLRMT (Fig. 6B), TFAM (Fig. 6C), and TFB2M (Fig. 6D) to the D-loop region was significantly reduced in HMGCS1-knockout cells compared to wild-type controls. This decrease in binding was consistent across all five D-loop regions examined, indicating that HMGCS1 deficiency impairs the recruitment or stability of the entire transcription machinery at the mitochondrial promoter regions. Collectively, these results demonstrate that HMGCS1 facilitates mitochondrial transcription by promoting the assembly of the transcription complex at the regulatory D-loop region.

In line with these findings, a direct comparison between parental and cisplatin-resistant cells using MtDIP revealed a significantly increased binding of not only HMGCS1 but also the core transcription factors POLRMT, TFAM, and TFB2M to the D-loop in the resistant cell line (Supplementary Fig. S3). This result further strengthens the correlation between the resistant state, elevated HMGCS1 levels, and the enhanced assembly of the mitochondrial transcription machinery at the mtDNA promoter.

### Pharmacological inhibition of HMGCS1 or mitochondrial transcription resensitizes cisplatin-resistant cervical cancer cells

Having established the role of mitochondrial HMGCS1 in promoting cisplatin resistance through enhanced mitochondrial transcription, we sought to determine whether pharmacological targeting of this pathway could restore drug sensitivity in resistant cells. To this end, we evaluated the effects of hymeglusin, a specific HMGCS1 inhibitor [34, 35], and IMT1B, a mitochondrial transcription inhibitor [36], on cisplatin resistance in cervical cancer cells.

**Fig. 7:**
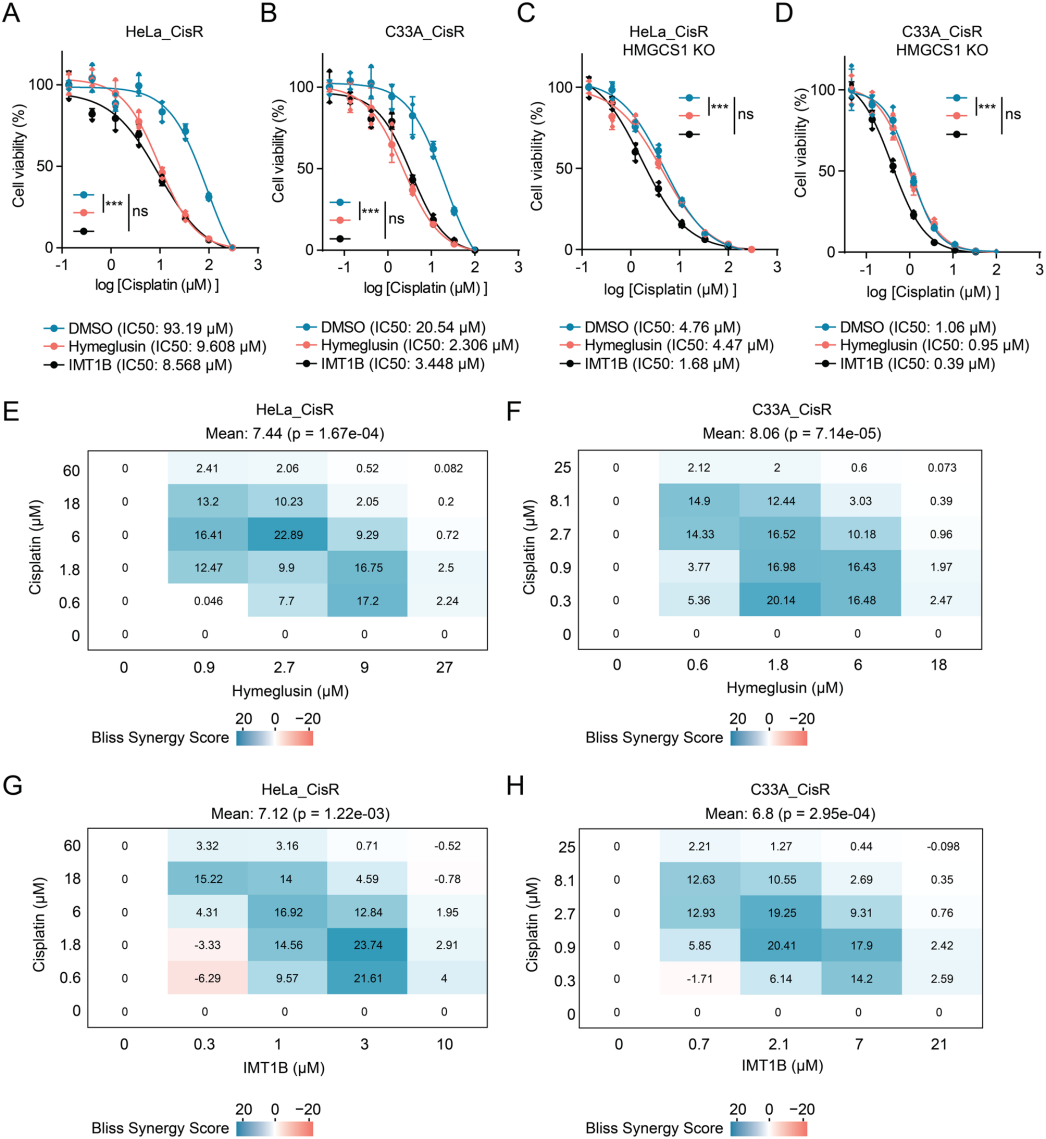
HMGCS1 inhibitor and mitochondrial transcription inhibitor synergize with cisplatin in drug-resistant cervical cancer cells. (**A**-**D**) Dose-response curves showing cell viability of cisplatin-resistant cells treated with increasing concentrations of cisplatin alone (DMSO control) or in combination with 2 µM hymeglusin or 1 µM IMT1B. Curves are shown for parental HeLa_CisR (**A**) and C33A_CisR (**B**) cells, and for their HMGCS1 knockout (KO) counterparts, HeLa_CisR HMGCS1 KO (**C**) and C33A_CisR HMGCS1 KO (**D**). IC50 values are indicated for each treatment condition. Statistical significance was determined by pairwise comparison of the LogIC50 value for each construct against the EV control using the Extra sum-of-squares F-test, with a Bonferroni correction for multiple comparisons (***p < 0.001, ns: not significant). (**E**-**H**) Synergy analysis of drug combinations in resistant cells using the Bliss independence model. Heat maps show synergy scores for various concentration combinations of cisplatin with hymeglusin in (**E**) HeLa_CisR and (**F**) C33A_CisR cells, or with IMT1B in (**G**) HeLa_CisR and (**H**) C33A_CisR cells. Positive values (blue) indicate synergistic interactions, values near zero (white) indicate additive effects, and negative values (red) indicate antagonistic interactions

First, we examined the impact of these inhibitors on the viability of cisplatin-resistant HeLa_CisR and C33A_CisR cells. The results showed that both hymeglusin and IMT1B dramatically reduced the IC50 values of cisplatin in these resistant cells (Fig. 7A-B), demonstrating that inhibition of either HMGCS1 or mitochondrial transcription can effectively reverse cisplatin resistance.

To further confirm that hymeglusin’s effect is mediated specifically through HMGCS1 and to delineate the hierarchical relationship within this resistance pathway, we conducted the same cisplatin sensitivity assays in HMGCS1 KO cisplatin-resistant cells. As expected, co-treatment with hymeglusin did not provide any additional sensitization to cisplatin in HMGCS1-KO cells (Fig. 7C-D). This result validates that the sensitizing effect of hymeglusin is strictly dependent on the presence of its target, HMGCS1. In contrast, the mitochondrial transcription inhibitor IMT1B significantly enhanced cisplatin sensitivity even in the absence of HMGCS1, causing a further reduction in IC50 values (Fig. 7C-D). This indicates that HMGCS1 functions as an upstream regulator of mitochondrial transcription, and that inhibiting the remaining basal mitochondrial transcription in HMGCS1-deficient cells can further augment cisplatin cytotoxicity. Collectively, these pharmacological experiments confirm that the HMGCS1-mitochondrial transcription axis is a critical driver of cisplatin resistance and a viable therapeutic target.

To assess potential synergistic effects between these inhibitors and cisplatin, we performed drug combination studies across a range of concentrations in cisplatin-resistant cervical cancer cells. Synergy was quantified using the Bliss independence model, where positive scores indicate synergistic interactions. The results showed that both hymeglusin and IMT1B exhibited significant synergy with cisplatin in cisplatin-resistant cervical cancer cells (Fig. 7E-H). These findings indicate that pharmacological inhibition of either HMGCS1 or mitochondrial transcription can effectively sensitize cisplatin-resistant cervical cancer cells to cisplatin treatment. The observed synergistic interactions suggest that combining these inhibitors with cisplatin may provide a promising therapeutic strategy to overcome drug resistance in cervical cancer.

In summary, our findings uncover a critical role for mitochondrial HMGCS1 in driving cisplatin resistance through enhanced mitochondrial transcription and biogenesis in cervical cancer. Importantly, pharmacological inhibition of either HMGCS1 or mitochondrial transcription effectively resensitized resistant cells to cisplatin treatment, with strong synergistic effects observed in combination therapy. These results identify the HMGCS1-mitochondrial transcription axis as a promising therapeutic target to overcome cisplatin resistance in cervical cancer patients.

## Discussion

This study reveals a novel regulatory role for HMGCS1 in mediating cisplatin resistance in cervical cancer through its mitochondrial localization and regulation of mitochondrial transcription. Our findings identify an unprecedented function of HMGCS1 as a non-canonical regulator of mitochondrial gene expression and establish a mechanistic connection between this metabolic enzyme and chemoresistance.

The subcellular compartmentalization of HMGCS1 represents a critical determinant of its function in chemoresistance. While canonically recognized as a cytosolic enzyme in the mevalonate pathway, our previous investigations identified HMGCS1 in both nuclear and mitochondrial compartments in cervical cancer cells [16]. The present study advances this understanding by demonstrating that mitochondrial, but not nuclear, HMGCS1 promotes cisplatin resistance. These data underscore the importance of examining protein subcellular distribution when investigating mechanisms of drug resistance. Given that metabolic reprogramming and reliance on mitochondrial function are common features across many malignancies, it is plausible that the chemoresistance-promoting function of mitochondrial HMGCS1 is not restricted to cervical cancer but represents a more general mechanism of chemoresistance. Future studies are needed to explore the generalizability of this finding in other malignancies.

Our findings strongly suggest that enhanced mitochondrial respiratory capacity, mediated by mitochondrial HMGCS1, is a key feature contributing to cisplatin resistance in cervical cancer cells. Cisplatin-resistant cells exhibited significantly elevated basal and maximal respiration compared to parental counterparts, with this enhanced respiratory phenotype dependent on HMGCS1 expression. Mitochondrial respiration has been implicated in chemoresistance across multiple malignancies, including ovarian, lung, and breast cancers [37–39]. Enhanced oxidative phosphorylation may contribute to drug resistance through multiple mechanisms: increased ATP production supporting energy-dependent processes such as drug efflux, reduced ROS generation due to efficient electron transport, and altered mitochondrial membrane potential affecting apoptotic signaling [40–44]. Our findings identify HMGCS1 as a critical regulator of mitochondrial respiratory capacity in cisplatin-resistant cervical cancer cells. While this study focused on cisplatin, the mechanism we have identified—enhancement of mitochondrial respiration to manage cellular stress—is a fundamental pro-survival strategy. Therefore, it is conceivable that mitochondrial HMGCS1 may confer resistance to a broader range of chemotherapeutic agents that induce apoptosis via mitochondrial pathways or impose significant oxidative stress, such as other platinum-based drugs or anthracyclines. Future studies are warranted to explore this possibility and determine the full spectrum of chemoresistance mediated by this pathway.

The identification of HMGCS1 as a regulator of mitochondrial transcription represents the most significant finding of this investigation. We demonstrated that HMGCS1 physically associates with the mitochondrial D-loop region containing the principal regulatory elements for mtDNA transcription. HMGCS1 deficiency reduced the binding of core transcription machinery components—POLRMT, TFAM, and TFB2M—to the D-loop region, resulting in decreased synthesis of mtDNA-encoded transcripts without affecting their stability. These data establish HMGCS1 as a non-canonical regulator of mitochondrial gene expression, representing a significant advancement in understanding the regulation of mitochondrial function in cancer cells.

The molecular mechanism by which HMGCS1 facilitates assembly of the mitochondrial transcription machinery requires further elucidation. Several hypotheses warrant investigation: (1) HMGCS1 may directly interact with transcription machinery components to stabilize their binding to mtDNA; (2) HMGCS1 might modify the local nucleoid structure to promote accessibility of the D-loop region; or (3) HMGCS1 could influence metabolite levels that affect mitochondrial transcription factor activity or binding. Future investigations employing proteomics, metabolomics, and structural biology approaches will elucidate these mechanistic details.

Lipid metabolic reprogramming is increasingly recognized as a hallmark of cancer that contributes to malignant phenotypes, including drug resistance [45]. In this context, the elevated cholesterol levels observed in cisplatin-resistant cells suggest potential involvement of HMGCS1’s canonical metabolic function in resistance. Cholesterol constitutes an essential component of mitochondrial membranes and influences membrane fluidity, respiratory complex assembly, and mitochondrial membrane potential [46–49]. Recent investigations have demonstrated that mitochondrial cholesterol modulates apoptotic sensitivity by affecting mitochondrial membrane permeability [50]. Therefore, HMGCS1 may contribute to cisplatin resistance through dual mechanisms: direct regulation of mitochondrial transcription and enhancement of cholesterol biosynthesis to support mitochondrial membrane integrity and function.

Our findings present significant therapeutic implications. The synergistic effect observed when combining cisplatin with either HMGCS1 inhibition (hymeglusin) or mitochondrial transcription inhibition (IMT1B) reveals promising strategies to overcome cisplatin resistance in cervical cancer. This approach aligns with emerging strategies targeting mitochondrial metabolism to enhance chemotherapy efficacy. However, clinical translation of these findings necessitates careful evaluation of potential adverse effects associated with inhibiting mitochondrial function in normal tissues.

Several limitations of this study should be addressed in future investigations. First, while we established a clear association between mitochondrial HMGCS1 and cisplatin resistance in vitro, validation in patient-derived models and clinical specimens is essential to confirm clinical relevance. Second, the potential interplay between HMGCS1’s role in mitochondrial transcription and its canonical function in cholesterol biosynthesis requires further examination. Finally, the precise stoichiometry and structural basis of HMGCS1’s interaction with mtDNA and the mitochondrial transcription machinery remain to be determined.

In conclusion, this study identifies mitochondrial HMGCS1 as a novel regulator of mitochondrial transcription and a key mediator of cisplatin resistance in cervical cancer. By facilitating assembly of the transcription machinery at the mtDNA D-loop region, HMGCS1 enhances expression of respiratory complex components, thereby promoting mitochondrial respiratory capacity and drug resistance. These findings expand our understanding of non-canonical functions of metabolic enzymes in cancer and establish the HMGCS1-mitochondrial transcription axis as a potential therapeutic target to overcome chemoresistance in cervical cancer.

## Supplementary Information

Below is the link to the electronic supplementary material.

Supplementary Material 1: Supplementary Fig. S1. HMGCS1 status and subcellular localization do not affect the basal proliferation of cervical cancer cells. (A-B) Proliferation curves of wild-type (WT) and HMGCS1 knockout (KO) HeLa (A) and C33A (B) cells. (C-D) Western blot analysis confirming comparable expression levels of 6xHis-tagged constructs in total cell lysates of HeLa (C) and C33A (D) cells expressing empty vector (EV), mitochondria-targeted HMGCS1 (MTS), or nuclear-localized HMGCS1 (NLS). TUBA1A serves as a loading control. (E) Western blot analysis of subcellular fractions (nuclei, cytosol, mitochondria) from HeLa cells expressing the constructs. The 6xHis tag confirms the specific localization of NLS-HMGCS1 to the nucleus and MTS-HMGCS1 to the mitochondria, with minimal cytosolic mislocalization. Lamin B1, TUBA1A, and Tomm20 serve as markers for nuclear, cytosolic, and mitochondrial fractions, respectively. (F-I) Proliferation curves of wild-type (F, G) and HMGCS1-KO (H, I) cells expressing EV, MTS, or NLS constructs. Cell proliferation was measured over a 5-day period using the SRB assay. Data for proliferation curves are presented as mean ± SD. ns: not significant, by Two-way ANOVA

Supplementary Material 2: Supplementary Fig. S2. Cisplatin-resistant cells exhibit enhanced mitochondrial RNA synthesis without changes in RNA stability. (A-F) RNA synthesis assays measuring the relative accumulation of newly synthesized transcripts in parental and cisplatin-resistant (CisR) HeLa cells at an initial (T0) and 2-hour (T1) time point. The analysis includes mtDNA-encoded genes (MT-ND1, MT-ND5, MT-CO1, MT-CYB, and MT-ATP6) and the nuclear-encoded 18 S rRNA control. (G-L) RNA decay assays measuring the stability of the same transcripts in parental and CisR cells following transcription inhibition. RNA levels were measured immediately (T0) and 24 h (T1) after inhibitor treatment. Data are presented as mean ± SD. Statistical significance was determined using Student’s t-test: *p < 0.05, **p < 0.01, ***p < 0.001, ns: not significant

Supplementary Material 3: Supplementary Fig. S3. Cisplatin-resistant cells show increased binding of HMGCS1 and core transcription factors to the MtDNA D-loop. (A-D) Mitochondrial DNA immunoprecipitation (MtDIP) analysis showing the binding of HMGCS1 (A), mitochondrial RNA polymerase (POLRMT) (B), mitochondrial transcription factor A (TFAM) (C), and mitochondrial transcription factor B2 (TFB2M) (D) to different regions of mtDNA in parental and cisplatin-resistant (CisR) HeLa cells. The binding enrichment is presented as a percentage of input. Data are presented as mean ± SD. Statistical significance was determined using Student’s t-test: *p < 0.05, **p < 0.01, ***p < 0.001, ns: not significant

Supplementary Material 4

Supplementary Material 5

## Data Availability

All data supporting the findings of this study are included in the manuscript and supplementary materials. Raw data files are available from the corresponding author upon reasonable request.
