# Supplementary figures and images for: Mitochondrial HMGCS1 mediates cisplatin resistance in cervical cancer through regulation of mitochondrial transcription
Source: BMC Mol Cell Biol. 2026 Jan 16;27:5. doi: 10.1186/s12860-026-00566-y (PMC12895831; doi:10.1186/s12860-026-00566-y)

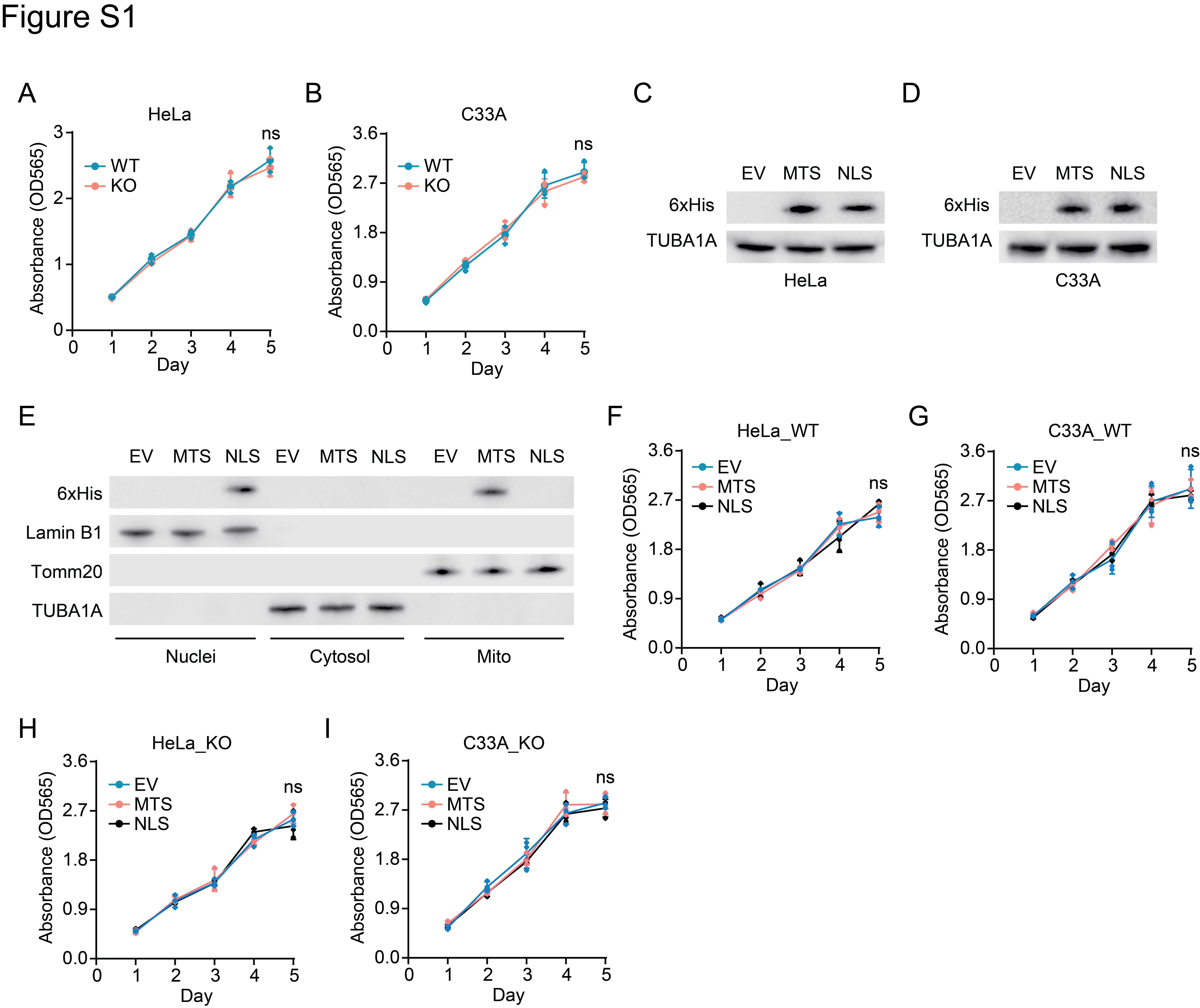

Supplement: Supplementary file 1 — Supplementary Material 1: Supplementary Fig. S1. HMGCS1 status and subcellular localization do not affect the basal proliferation of cervical cancer cells. (A-B) Proliferation curves of wild-type (WT) and HMGCS1 knockout (KO) HeLa (A) and C33A (B) cells. (C-D) Western blot analysis confirming comparable expression levels of 6xHis-tagged constructs in total cell lysates of HeLa (C) and C33A (D) cells expressing empty vector (EV), mitochondria-targeted HMGCS1 (MTS), or nuclear-localized HMGCS1 (NLS). TUBA1A serves as a loading control. (E) Western blot analysis of subcellular fractions (nuclei, cytosol, mitochondria) from HeLa cells expressing the constructs. The 6xHis tag confirms the specific localization of NLS-HMGCS1 to the nucleus and MTS-HMGCS1 to the mitochondria, with minimal cytosolic mislocalization. Lamin B1, TUBA1A, and Tomm20 serve as markers for nuclear, cytosolic, and mitochondrial fractions, respectively. (F-I) Proliferation curves of wild-type (F, G) and HMGCS1-KO (H, I) cells expressing EV, MTS, or NLS constructs. Cell proliferation was measured over a 5-day period using the SRB assay. Data for proliferation curves are presented as mean ± SD. ns: not significant, by Two-way ANOVA [file 12860_2026_566_MOESM1_ESM.png]

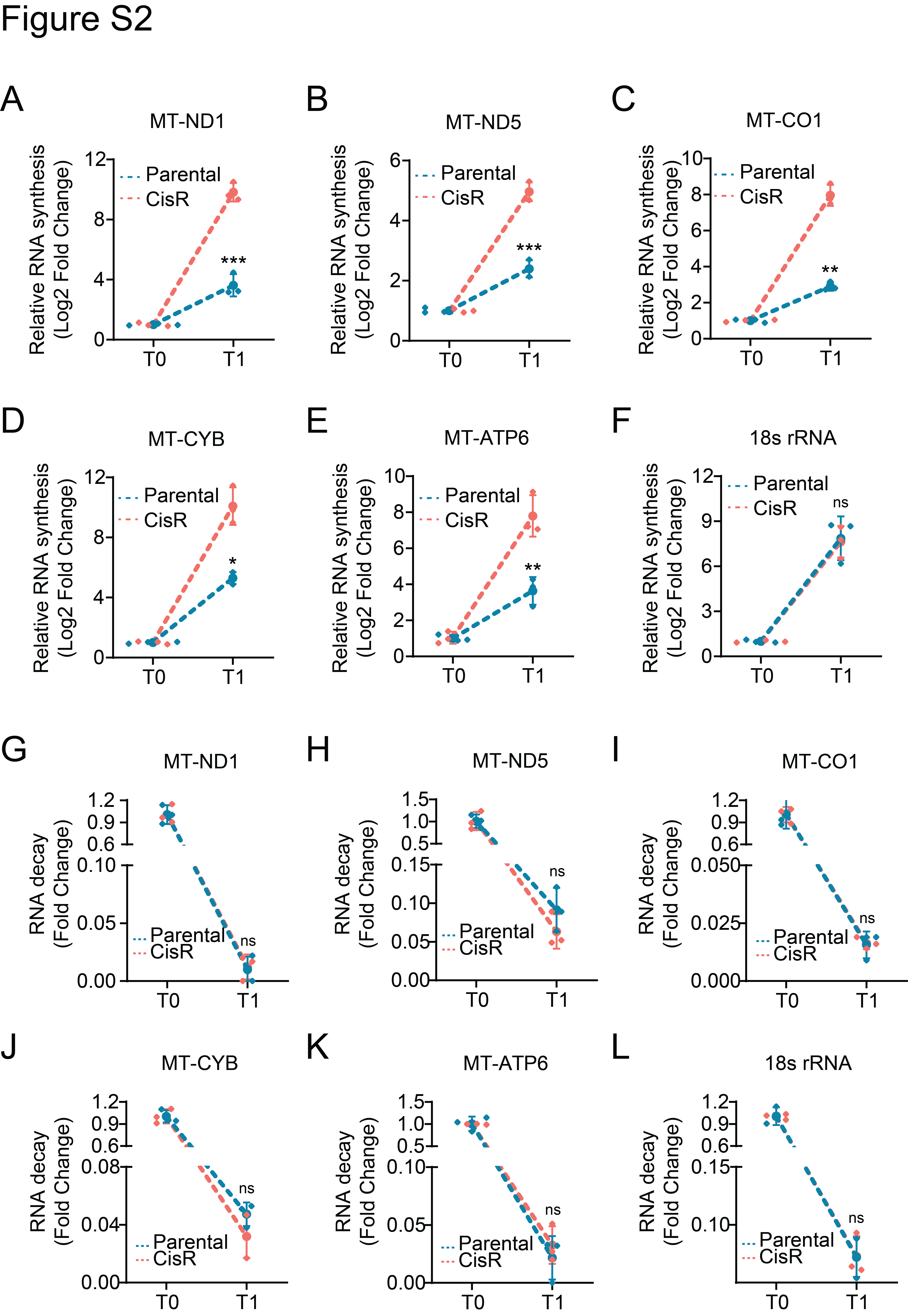

Supplement: Supplementary file 2 — Supplementary Material 2: Supplementary Fig. S2. Cisplatin-resistant cells exhibit enhanced mitochondrial RNA synthesis without changes in RNA stability. (A-F) RNA synthesis assays measuring the relative accumulation of newly synthesized transcripts in parental and cisplatin-resistant (CisR) HeLa cells at an initial (T0) and 2-hour (T1) time point. The analysis includes mtDNA-encoded genes (MT-ND1, MT-ND5, MT-CO1, MT-CYB, and MT-ATP6) and the nuclear-encoded 18 S rRNA control. (G-L) RNA decay assays measuring the stability of the same transcripts in parental and CisR cells following transcription inhibition. RNA levels were measured immediately (T0) and 24 h (T1) after inhibitor treatment. Data are presented as mean ± SD. Statistical significance was determined using Student’s t-test: *p < 0.05, **p < 0.01, ***p < 0.001, ns: not significant [file 12860_2026_566_MOESM2_ESM.png]

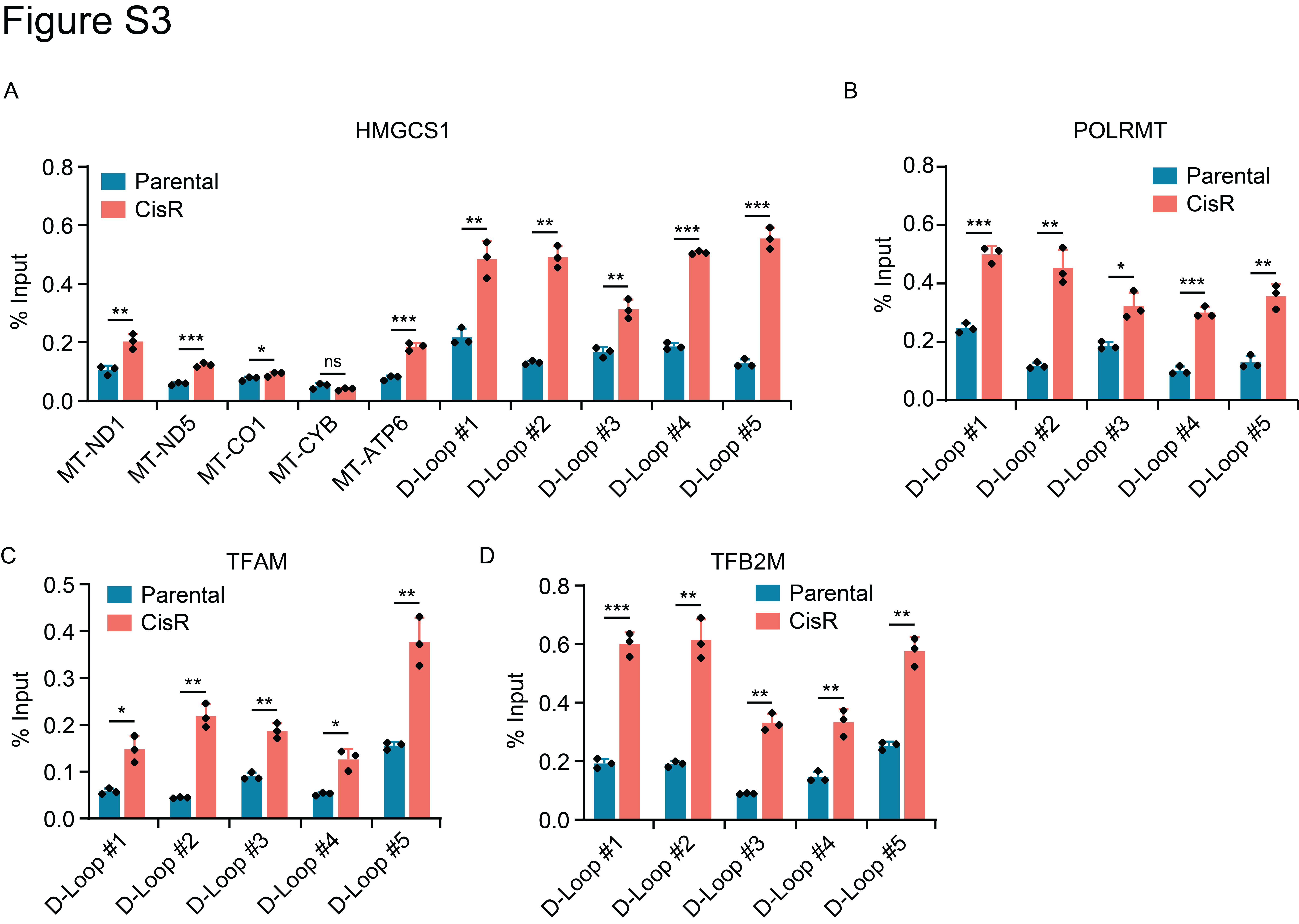

Supplement: Supplementary file 3 — Supplementary Material 3: Supplementary Fig. S3. Cisplatin-resistant cells show increased binding of HMGCS1 and core transcription factors to the MtDNA D-loop. (A-D) Mitochondrial DNA immunoprecipitation (MtDIP) analysis showing the binding of HMGCS1 (A), mitochondrial RNA polymerase (POLRMT) (B), mitochondrial transcription factor A (TFAM) (C), and mitochondrial transcription factor B2 (TFB2M) (D) to different regions of mtDNA in parental and cisplatin-resistant (CisR) HeLa cells. The binding enrichment is presented as a percentage of input. Data are presented as mean ± SD. Statistical significance was determined using Student’s t-test: *p < 0.05, **p < 0.01, ***p < 0.001, ns: not significant [file 12860_2026_566_MOESM3_ESM.png]

Figure 1A, 1B

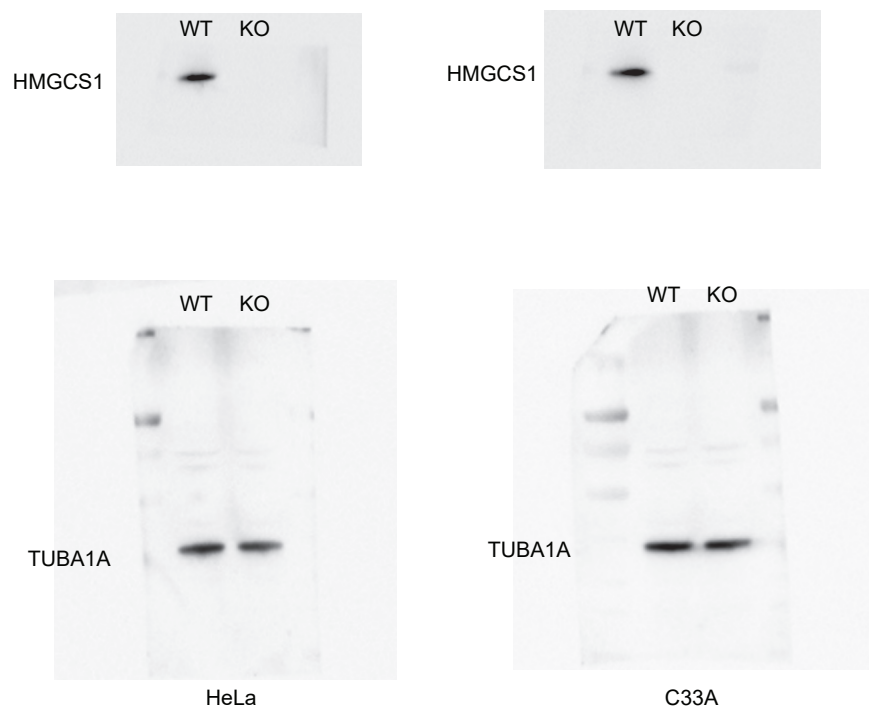

Figure S1C, S1D

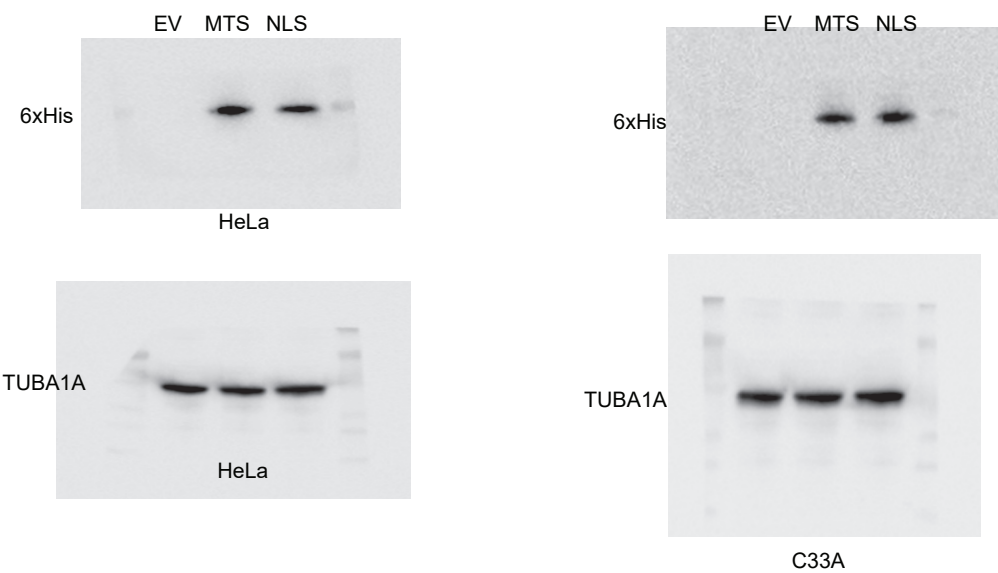

Figure 1E

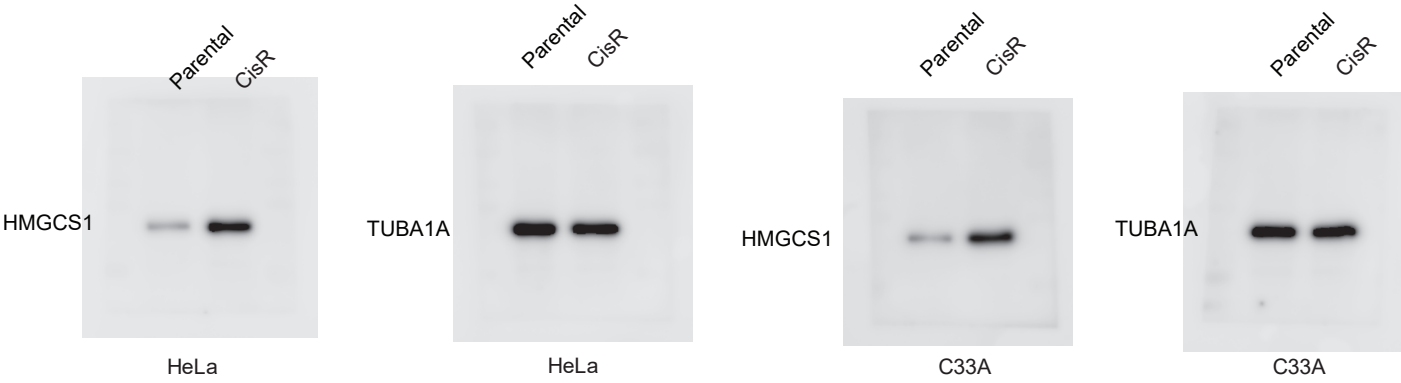

Figure 1H

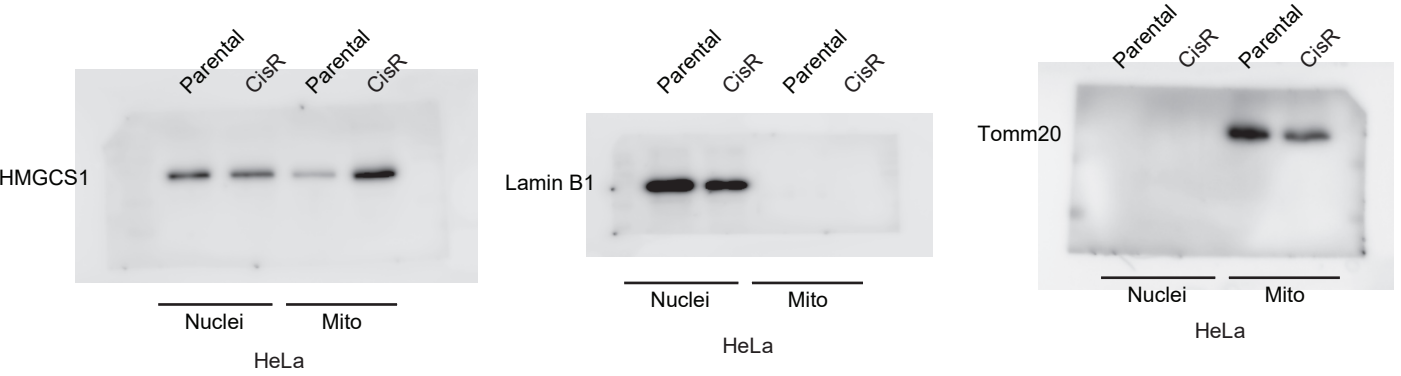

Figure 1I

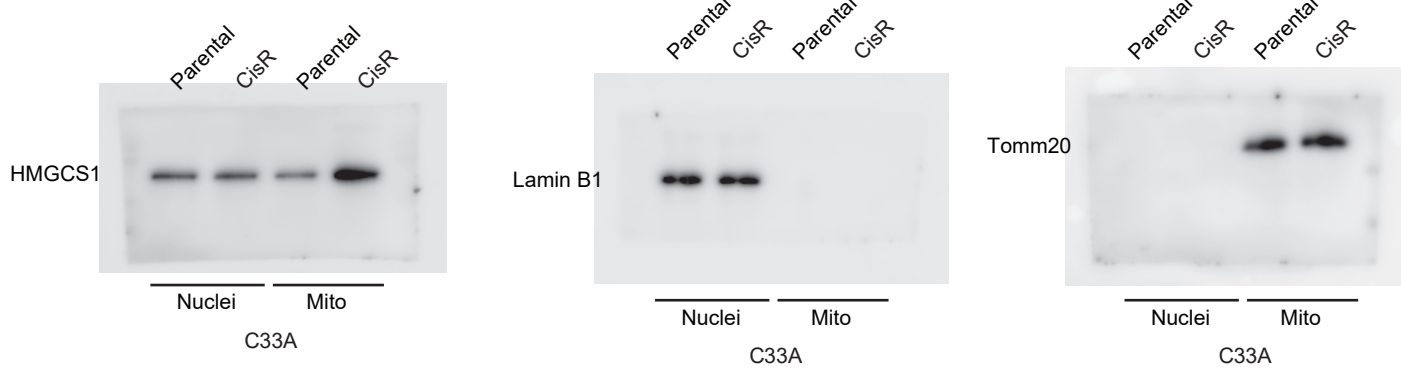

Figure S1E

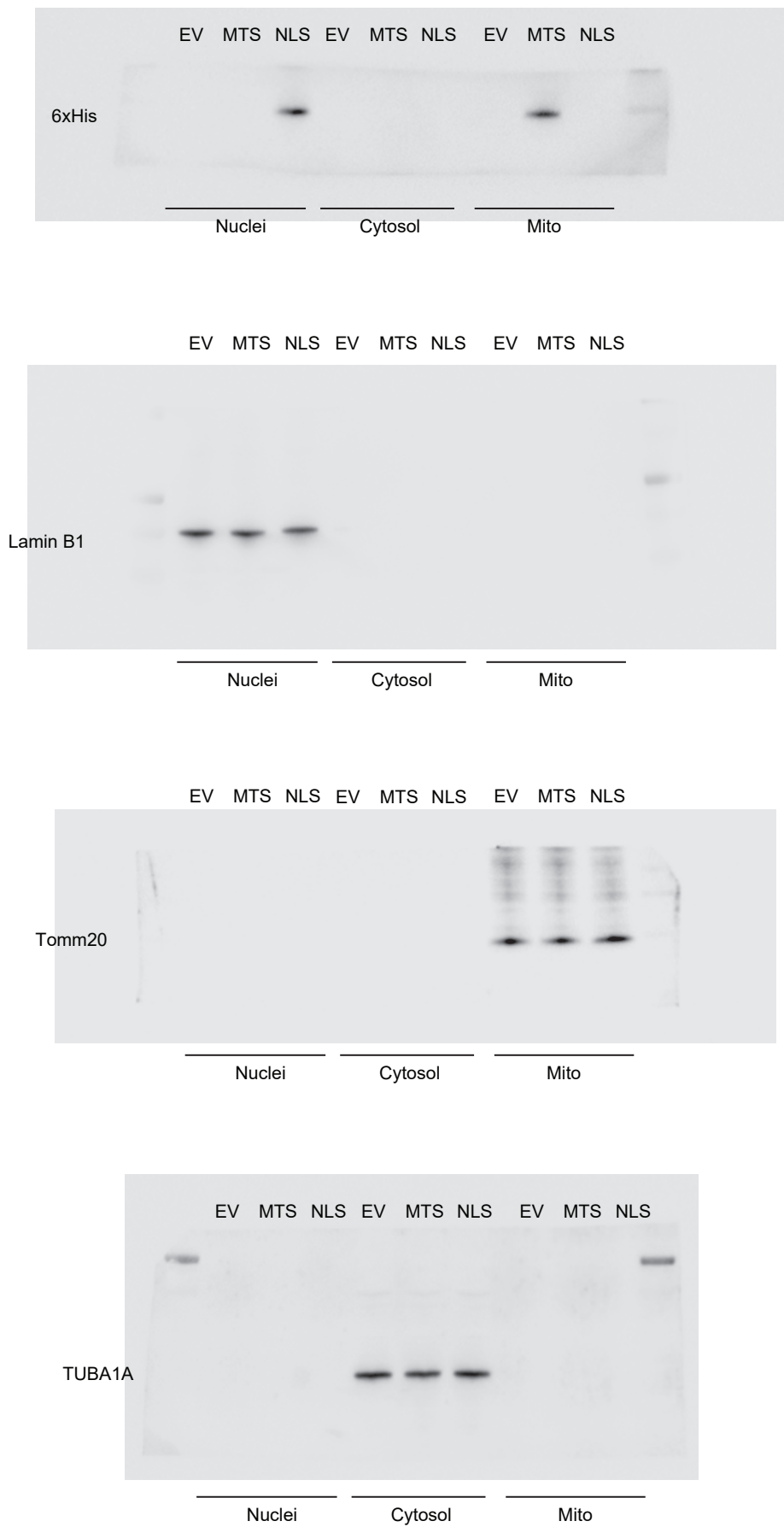

Supplement: Supplementary file 5 — Supplementary Material 5 [file 12860_2026_566_MOESM5_ESM.pdf]
