# Supplementary material for: Mitochondrial HMGCS1 mediates cisplatin resistance in cervical cancer through regulation of mitochondrial transcription
Source: BMC Mol Cell Biol. 2026 Jan 16;27:5. doi: 10.1186/s12860-026-00566-y (PMC12895831; doi:10.1186/s12860-026-00566-y)
Supplement: Supplementary file 4 — Supplementary Material 4 [file 12860_2026_566_MOESM4_ESM.docx]

Supplementary Table 1. Information regarding the primers used in this study

| Target | Forward (5’-3’) | Reverse (5’-3’) |
| --- | --- | --- |
| MT-ND1 | CCCTAAAACCCGCCACATCT | GAGCGATGGTGAGAGCTAAGGT |
| MT-ND5 | TCTTAGTTACCGCTAACAACC | ATAATTCCTACGCCCTCTCAG |
| MT-CO1 | ATATTTCACCTCCGCTACCA | TCAGCTAAATACTTTGACGCC |
| MT-CYB | ATCACTTTATTGACTCCTAGCC | TGGTTGTCCTCCGATTCAG |
| MT-ATP6 | TCCCTCTACACTTATCATCTTCAC | GACAGCGATTTCTAGGATAGTC |
| ACTB | GATTCCTATGTGGGCGACGA | TGTAGAAGGTGTGGTGCCAG |
| B2M | TGCTGTCTCCATGTTTGATGTATCT | TCTCTGCTCCCCACCTCTAAGT |
| D-loop #1 | CACCCCTCACCCACTAGGATAC | TCCATGGGGACGAGAAGGGATT |
| D-loop #2 | ATCACCCTATTAACCACTCACGG | GACATAGGGTGCTCCGGCTC |
| D-loop #3 | GAGCACCCTATGTCGCAGTAT | ACACTTTAGTAAGTATGTTCGCCT |
| D-loop #4 | GCTTCTGGCCACAGCACTTA | GTGCATACCGCCAAAAGATAAA |
| D-loop #5 | CCATTAGCACCCAAAGCTAAGA | CATAGCGGTTGTTGATGGGTG |
